# The social instability stress paradigm in rat and mouse: A systematic review of protocols, limitations, and recommendations

**DOI:** 10.1016/j.ynstr.2021.100410

**Published:** 2021-10-16

**Authors:** Amber Koert, Annemie Ploeger, Claudi L.H. Bockting, Mathias V. Schmidt, Paul J. Lucassen, Anouk Schrantee, Joram D. Mul

**Affiliations:** a Brain Plasticity Group, Swammerdam Institute for Life Sciences, Faculty of Science, University of Amsterdam, Amsterdam, the Netherlands; b Centre for Urban Mental Health, University of Amsterdam, Amsterdam, the Netherlands; c Department of Psychology, University of Amsterdam, Amsterdam, the Netherlands; d Amsterdam UMC, University of Amsterdam, Department of Psychiatry, Amsterdam, the Netherlands; e Max Planck Institute of Psychiatry, Research Group Neurobiology of Stress Resilience, Munich, Germany; f Amsterdam UMC, University of Amsterdam, Department of Radiology and Nuclear Medicine, Amsterdam, the Netherlands

**Keywords:** SIS, Social hierarchy, Depression, Anxiety, Social dominance, Rodents

## Abstract

**Background:**

Social stress is an important environmental risk factor for the development of psychiatric disorders, including depression and anxiety disorders. Social stress paradigms are commonly used in rats and mice to gain insight into the pathogenesis of these disorders. The social instability stress (SIS) paradigm entails frequent (up to several times a week) introduction of one or multiple unfamiliar same-sex home-cage partners. The subsequent recurring formation of a new social hierarchy results in chronic and unpredictable physical and social stress.

**Purpose:**

We compare and discuss the stress-related behavioral and physiological impact of SIS protocols in rat and mouse, and address limitations due to protocol variability. We further provide practical recommendations to optimize reproducibility of SIS protocols.

**Methods:**

We conducted a systematic review in accordance with the PRISMA statement in the following three databases: PubMed, Web of Science and Scopus. Our search strategy was not restricted to year of publication but was limited to articles in English that were published in peer-reviewed journals. Search terms included "social* instab*” AND ("animal” OR "rodent” OR "rat*” OR "mice” OR "mouse”).

**Results:**

Thirty-three studies met our inclusion criteria. Fifteen articles used a SIS protocol in which the composition of two cage mates is altered daily for sixteen days (SIS^16D^). Eleven articles used a SIS protocol in which the composition of four cage mates is altered twice per week for 49 days (SIS^49D^). The remaining seven studies used SIS protocols that differed from these two protocols in experiment duration or cage mate quantity. Behavioral impact of SIS was primarily assessed by quantifying depressive-like, anxiety-like, social-, and cognitive behavior. Physiological impact of SIS was primarily assessed using metabolic parameters, hypothalamus-pituitary-adrenal axis activity, and the assessment of neurobiological parameters such as neuroplasticity and neurogenesis.

**Conclusion:**

Both shorter and longer SIS protocols induce a wide range of stress-related behavioral and physiological impairments that are relevant for the pathophysiology of depression and anxiety disorders. To date, SIS^16D^ has only been reported in rats, whereas SIS^49D^ has only been reported in mice. Given this species-specific application as well as variability in reported SIS protocols, additional studies should determine whether SIS effects are protocol duration- or species-specific. We address several issues, including a lack of consistency in the used SIS protocols, and suggest practical, concrete improvements in design and reporting of SIS protocols to increase standardization and reproducibility of this etiologically relevant preclinical model of social stress.

## Highlights

• SIS is an etiologically relevant preclinical model of social stress.
• SIS induces a wide range of stress-related behavioral and physiological impairments.
• Variability in SIS protocols to date has produced inconsistent results.
• Establishment of a standardized SIS protocol is important to increase utility of this etiologically relevant stress model.

## 1 Introduction

### 1.1 Social behavior and mental health

Well-developed social behavior is considered essential for establishing and maintaining proper mental health. This can be illustrated by the psychological impact of the reduced social interactions during the COVID-19 pandemic. During periods of social isolation (*e*.*g*. quarantine), negative psychological effects are frequently reported (Brooks et al., 2020). Furthermore, a reduction in social contact is progressively associated with mental health impairments (Benke et al., 2020).

Social stress is also associated with the development of stress-related mental disorders (Mason et al., 2016). Many aspects of our modern society, such as high population density (*e*.*g*. in an urban environment), increase our frequency of social interactions. While a high number of social interactions can be beneficial and attractive for some, it may provoke social stress for others. Currently, the majority of the global human population lives in very dense urban areas (Zhang, 2016). As early as 1939, an ecological study addressed the adverse effects of the social (dis-)organization of a city, hinting at links to the increased prevalence of mental health disorders in urban communities (Faris and Dunham, 1939).

Taken together, disrupting, exceeding, or limiting social interactions can have negative effects on mental health and well-being. To better understand the mechanisms underlying these and related aspects of depression and/or anxiety, various rodent paradigms try to model alterations in social interaction dynamics in group-housed animals.

### 1.2 The social instability stress paradigm

Changing the dynamics of social interactions in a rodent paradigm can produce stress. Various examples of such models exist, including the social defeat, social isolation, and social instability stress (SIS) paradigms. In the social defeat paradigm, experimental animals are exposed to a dominant conspecific, that, via a combination of direct physical contact and indirect sensory contact, results in a stressful and subordinate relation (Golden et al., 2011; Rygula et al., 2005). Other stress-related rodent paradigms, such as the chronic unpredictable mild stress paradigm, induce stress by unpredictable alterations in the environment. These include exposure (for at least two weeks) to a variety of stressors in unpredictive order, such as water and/or food deprivation, social isolation or crowding, overnight/stroboscopic illumination, a tilted cage, white noise and/or wet bedding (Willner, 2017; Willner et al., 1992).

The SIS paradigm combines aspects of unpredictability and social stress and is based on frequent (daily to several times a week) alterations of the cage group composition, thereby exposing animals to unfamiliar same-sex cage partners on a regular basis. As the recurring formation of a new cage hierarchy induces social stress, the SIS paradigm is a unique paradigm with a high degree of face, construct and predictive validity (McCormick and Green, 2013; Scharf et al., 2013; Schmidt et al., 2008; Willner, 1984). Through its relative straight-forward design, SIS can be applied across social mammalian species, which allows for a better comparison between species, including humans, than other paradigms. Moreover, the SIS paradigm has been proposed to represent a valid model for (aspects of) social disorganization in urban areas (Tabibzadeh and Liisberg, 1997).

**Fig. 1:**
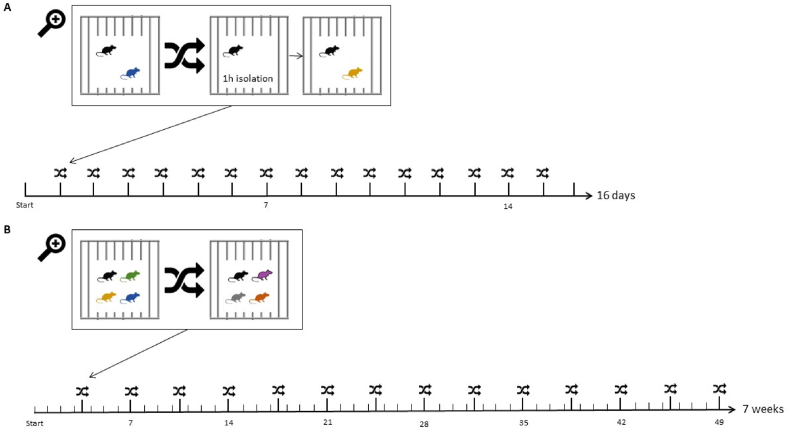
**Experimental timeline of SIS**^**16D**^**and SIS**^**49D**^**protocols.** (A) The SIS^16D^ protocol has a total duration of 16 days, during which the cage composition (two rodents/cage) is changed daily, often after a social isolation period of 1 h. (B) The SIS^49D^ protocol has a total duration of 49 days, during which the cage composition (four rodents/cage) is changed twice weekly, often without a social isolation period.

To date, two protocol variants of the SIS paradigm have been most frequently used in published literature (see Fig. 1). One variant lasts 16 days and the cage composition (two rodents/cage) is changed daily (hereafter referred to as SIS^16D^; see *e.g.* McCormick et al., 2007). Another commonly used SIS protocol lasts 49 days, and the group composition (four rodents/cage) is changed twice weekly (hereafter termed the SIS^49D^; see *e.g.* Schmidt et al., 2007). In both the SIS^16D^ and SIS^49D^ protocols, experimental animals are exposed to approximately the same number of alterations in cage composition (15 and 14 times, respectively). However, the experimental timeframe (16 versus 49 days) and the cage composition (two versus four animals) differ substantially between the protocols.

Other studies have used SIS protocols that vary slightly from the two above-mentioned protocols (see Appendix A and B). These studies have also been included in this systematic review. Although the concept of the SIS protocols is similar, large, and even small, differences in their experimental design could produce a substantial different impact on several stress-related parameters. A straightforward example of this is the rodent species used in the protocol, as rats have a very different natural social structure than mice. Although both rats and mice establish social hierarchies, rats are less territorial and aggressive than mice (Ellenbroek and Youn, 2016), with especially males differing strikingly in these behaviors (Blanchard et al., 2001). Therefore, similar SIS protocols may have different behavioral outcomes in rats and mice, or males and females.

### 1.3 Assessing the impact of SIS

The studies included in this review often probe the effects of SIS on overall behavioral performance by using several types of behavioral tests. To provide more definitive evidence for the presence of a depressive-like state, generally, a combination of emotional symptoms (anhedonia), homeostatic symptoms (sleep, appetite, body weight), psychomotor symptoms (locomotor activity, immobility, and explorative behavior), and impaired cognitive/social behavior should be measured. Moreover, compiling assessment from multiple tests, preferably that depend on different behavioral or emotional states (*e*.*g*. motor versus affective), into a Z score, instead of relying on one test or multiple parameters from the same test, will help provide more conclusive insight regarding the presence of a depressive-like state (Ritov et al., 2016).

Anhedonia, a core symptom of depression, can be assessed in rodents using the sucrose preference test (SPT; alternatively, the sweetener saccharin can be used as a non-caloric alternative), the social interaction test, or by quantifying sexual behavior (Nestler and Hyman, 2010). Locomotor activity can be assessed in a familiar environment (*e*.*g*. home-cage) or in a novel environment [*e.g.* the open field test (OFT)]. To assess adaptive behavior in response to acute stress, the forced swim test (FST) and tail suspension test (TST) can be used (Nestler and Hyman, 2010). Anxiety-like behavior can be assessed using the elevated plus maze (EPM) and the light-dark box test. Cognitive behavior is generally assessed with the Morris water maze (MWM), object recognition and location, and fear conditioning (Belzung and Griebel, 2001).

Physiological parameters following SIS can be scored throughout the entire study and can often be scored in a minimally invasive or stressful manner. Physiological symptoms of a depressive- or anxiety-like state include changes in body weight and changes in locomotor activity. In addition, fur condition can be scored by an observer and is considered indicative of the animal's well-being, with piloerection, impoverished fur condition, or decreased grooming latency occurring during a depressive-like state (Ducottet et al., 2003; Santarelli et al., 2003). Another common physiological indication of stress is blood corticosterone (CORT) dynamics, with blood collected via tail sampling. Physiological impact of SIS is also determined after sacrifice of the experimental animals and includes the assessment of changes in gene and protein expression in various tissues, including the brain.

In this systematic review we assess the impact of SIS protocols on several of these stress-related, behavioral, and physiological parameters in rats and mice. We also discuss the role of species-, sex-, frequency-, and duration-related effects on experimental results and how this impacts applicability of the various SIS protocols for stress-related studies.

## 2 Methods/design

We performed a systematic review in accordance with the guidelines from The Preferred Reporting Items for Systematic Reviewed and Meta-analyses (PRISMA) Statement (Moher et al., 2009).

### 2.1 Search strategy

**Fig. 2:**
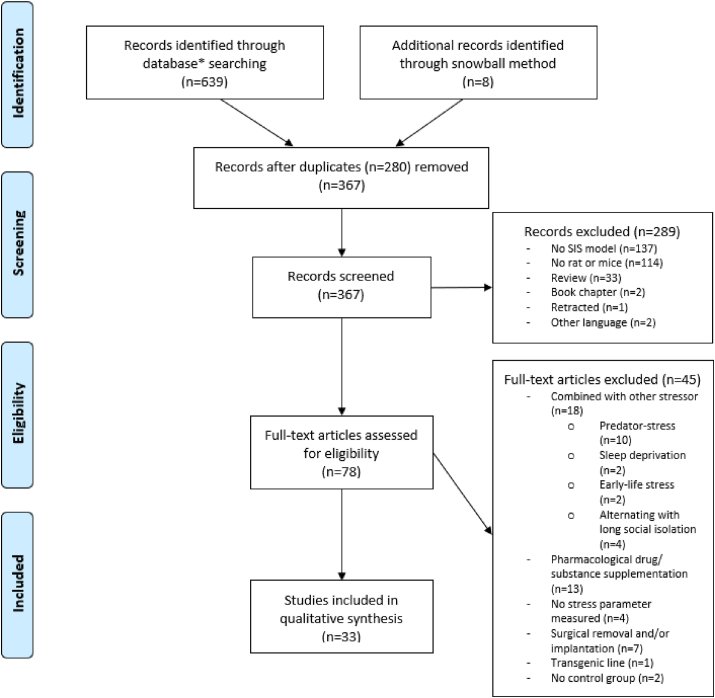
**PRISMA flow diagram of article selection process.** *databases of PubMed, Web of Science and Scopus were consulted.

An electronic literature search of peer-reviewed journal articles was conducted between May 2020 and December 2020 using three databases (PubMed, Web of Science and Scopus). Additionally, the snowball method was used to review the references of the retrieved articles and identify other eligible studies which initially did not appear from the database searches. The search was not restricted to year of publication but limited to peer-reviewed original research articles with full text available published in English. Reviews, meta-analyses and other types of articles (*e*.*g*. book chapters, retracted articles) were excluded. Titles, abstracts and methods were screened by the lead author (A.K.) for relevance based on the selection criteria (Section 2.2) and all duplicates were removed. The relevant articles were selected for further consideration (Fig. 2).

### 2.2 Search terminology, selection criteria and data extraction

The search terms used included: "social* instab*” AND ("animal” OR "rodent” OR "rat*” OR "mice” OR "mouse”). Articles were eligible for inclusion when the article: (1) included a SIS paradigm; (2) investigated non-transgenic rats or mice; (3) assessed the impact of SIS on stress-related behavioral and/or physiological parameters; (4) included a healthy control group. Consequently, studies were excluded when (A) the SIS stressor was combined with a second stressor; (B) solely focused on the effects of a pharmacological drug or supplementation of other substances; (C) had surgical removal of the ovaries or vasectomy and/or (CORT) implants (see Supplementary Data). Upon completion of title and abstract screening, a total of 74 articles were identified for full-text review. Reasons for exclusion were recorded for each article.

### 2.3 Data analysis

After study selection, criteria were followed to maintain the evaluation of the studies within narrow standards. The first and essential criterion was that all details regarding the SIS protocol were described, along with a detailed description of experimental testing methods and timing.

The following data were extracted: author and year of publication; animal species, strain and sex; number of animals in experimental- and control group; age during SIS protocol; SIS protocol; timing of behavioral/physiological assessments; behavioral outcome measurements; physiological outcome measurements.

## 3 Results

### 3.1 Study inclusions

Of all assessed articles, thirty-three articles met the inclusion criteria, and these articles were published between 2007 and 2020 (see Appendix A and B for a detailed overview of the characteristics and results of all studies).

### 3.2 SIS protocols and experimental animal characteristics

Nineteen articles assessed the effect of SIS in rats and fourteen articles assessed the effect of SIS in mice, with total sample size ranging from 16 to 200 experimental animals (see Appendix A and B, respectively). The SIS^16D^ protocol has uniquely been reported in rats and was applied to Long-Evans rats in 14 articles and to Sprague-Dawley rats in one article (15 articles total; see Appendix A). The SIS^49D^ protocol has uniquely been reported in mice and was applied to CD1 mice in nine articles and to C57BL/6 mice in two articles (11 articles total; see Appendix B). The remaining seven articles applied alternative SIS protocols to Sprague-Dawley or Wistar rats, or to Balb/c, SJL or CD1 mice (see Appendix A and B, respectively). The shortest SIS protocol lasted 11 days and cage composition of two mice per cage was changed daily (de Lima and Massoco, 2017). Another SIS protocol lasted 19 days and cage composition of two mice per cage was also changed daily (Chatterjee et al., 2009). Two studies applied a 28-day SIS protocol to three animals per cage, and cage composition of mice was changed daily (Dadomo et al., 2018) and cage composition of rats was changed three times a week (Pittet et al., 2017). Another SIS protocol lasted 35 days and cage composition of five rats per cage was changed daily (Tsai et al., 2014). One study changed cage composition of six rats per cage three times per week, and SIS lasted either 35 days or 100 days (Eskandari Sedighi et al., 2015). Lastly, a SIS protocol was applied to ten rats per cage, and cage composition was changed daily for 42 days (Maslova et al., 2010). Animals in the control group are commonly housed with the same number of cage mates as the SIS group, but in a consistent manner (*i*.*e*. without the frequent cage mate rotation), and sometimes with provision of a clean cage at the same frequency as the SIS group.

The age of the rodents at the start of SIS protocols ranged from postnatal day (PND) 21 (*i*.*e*. adolescence) to PND84 (*i*.*e*. adulthood). In addition, the timing of the behavioral and physiological assessments varied. Some studies assessed the immediate consequences of SIS by measuring the behavioral and/or physiological parameters during or shortly after the protocol (see Appendix A and B). Other studies focused on the long-lasting consequences of the protocol, in which behavioral and/or physiological parameters were assessed weeks, months, or even one year after the end of the SIS protocol (see Appendix A and B).

Of all 33 reviewed articles, 21 articles studied males, five articles studied females and seven articles studied both sexes.

### 3.3 Behavioral impact of SIS

#### 3.3.1 Depressive-like behavior

The development of anhedonia, a core symptom of human depression, is commonly assessed in rodents using the SPT (Nestler and Hyman, 2010). To assess stress coping mechanisms in rodents, the FST and TST can be used to score adaptive behavioral responses to an inescapable stressor (Molendijk and de Kloet, 2015).

In general, mice appear more susceptible to develop depressive-like behavior after exposure to SIS than rats. Notably, depressive-like behavior in response to SIS seems to develop independently of the duration of the SIS paradigm. For example, 11-day SIS applied to adolescent male Balb/c mice increased immobile behavior during the FST when tested 20 days later during the late light phase (de Lima and Massoco, 2017).

Twenty-eight-day SIS applied to adult female Sprague-Dawley rats did not affect saccharin preference during the dark phase when tested seven days following SIS termination (Pittet et al., 2017). Likewise, SIS^16D^ applied to adolescent male Long-Evans rats did not affect sucrose preference when tested immediately or 21 days following SIS termination (Marcolin et al., 2019). However, SIS^16D^ increased sucrose solution consumption when rats had to compete for limited sucrose access with a cage mate, and this competitive preference occurred independent of the age tested (Marcolin et al., 2019). Furthermore, 42-day SIS applied to adolescent male Wistar rats did not affect immobile behavior in the FST when tested immediately or 70 days following SIS termination (Maslova et al., 2010).

SIS^49D^ applied to adolescent male C57BL/6N mice decreased sucrose preference when tested after 12 months of individual housing (Wang et al., 2019), indicative of the development of anhedonia (Goshen et al., 2008; Rygula et al., 2005). Twenty-eight-day SIS applied to adult female CD1 mice decreased sucrose preference when tested at 2, 14, and 28 days following onset of SIS (Dadomo et al., 2018). SIS^49D^ applied to adult male and female C57BL/6J mice did not alter immobile behavior during the FST when tested 34 days following SIS termination (Yohn et al., 2019). SIS^49D^ applied to adolescent male C57BL/6N mice decreased latency to immobile behavior, but did not alter the total duration of immobile behavior, during the TST, when tested after 12 months of individual housing (Wang et al., 2019). Eleven-day SIS applied to adolescent male Balb/c mice decreased latency to immobile behavior and increased total duration of immobile behavior during the TST when tested 20 days following SIS and during the late light phase (de Lima and Massoco, 2017). Finally, SIS^49D^ applied to adolescent male CD1 mice increased immobile behavior during the TST when tested 35 days following SIS and during the early light phase, but only in the vulnerable, and not resistant, SIS subgroup (Schmidt et al., 2010b).

#### 3.3.2 Anxiety-like behavior

Anxiety-like behavior is commonly assessed using the EPM, OFT or light-dark box test. These tests can visualize alterations in locomotor activity, explorative behavior, and risk-taking behavior. The latter can also be assessed using the novelty suppressed feeding test, in which the latency to eat familiar food in an aversive novel environment is indicative for anxiety (Samuels and Hen, 2011). In addition, consumption of ethanol has anxiolytic properties and can as such be used as a measure for anxiety-related behavior (Spanagel et al., 1995).

In general, SIS applied to rats has resulted in inconsistent results, with SIS increasing, decreasing, or not affecting anxiety-related measures. Conversely, SIS applied to mice generally increased or did not change anxiety-like behavior compared to non-stressed controls.

In male adolescent Long-Evans rats or male Wistar rats, both SIS^16D^ and six-week SIS did not affect the total time spent in the open arms of an EPM, a behavior that is indicative of risk-taking and explorative behavior, when measured immediately, three or ten weeks following SIS (Hodges et al., 2018; Marcolin et al., 2020; Maslova et al., 2010; Roeckner et al., 2017). In female adolescent Long-Evans rats, SIS^16D^ decreased the total time spent in the open arms of an EPM when measured three days following SIS (Roeckner et al., 2017), whereas another study reported increased total time spent in the open arms of an EPM when measured immediately following SIS^16D^ (McCormick et al., 2008). In adolescent male Long-Evans rats, SIS^16D^ reduced the latency to enter the center during an OFT when measured 3.5 weeks following SIS (Green et al., 2013). In adolescent male Long-Evans rats, SIS^16D^ increased 10% alcohol intake when measured immediately following SIS, irrespective of the social context during the ethanol intake test, but these effects were minimal when measured again weeks later during adulthood (Marcolin et al., 2019). Another study from the same group reported no changes in sweetened 10% alcohol intake during intermittent access to ethanol during three weeks following SIS^16D^ in adolescent male Long-Evans rats (Marcolin et al., 2020). In a different laboratory, SIS^16D^ applied to adolescent male or female Long-Evans rats also did not impact ethanol intake at various timepoints following SIS (Roeckner et al., 2017). Nonetheless, in this study, SIS^16D^ increased preference for ethanol over water in male rats, an effect that was not observed in stressed female rats (Roeckner et al., 2017).

In adult female CD1 mice, adolescent male and female CD1 mice, and adult male and female C57BL/6J mice, both relative short and long SIS protocols did not affect total time spent in the open arms of the EPM when tested during or at various timepoints following SIS (Dadomo et al., 2018; Scharf et al., 2013; Schmidt et al., 2007, 2010a, 2010b; Sterlemann et al., 2008; Yohn et al., 2019). In contrast, and using a slightly different experimental design with SIS mice ranging from three to five per cage, SIS^49D^ applied to adult female C57BL/6J mice travelled less on the open arms of the EPM when tested four weeks following SIS (Yohn et al., 2019).

Finally, SIS^49D^ applied to male and female CD1 mice decreased time spent on the open arms of the EPM in stressed females, but not stressed males, compared to non-stressed controls when tested two months following SIS (Saavedra-Rodríguez and Feig, 2013). SIS^49D^ applied to adult male and female C57BL/6J mice reduced distance travelled in the light compartment in the light-dark box test, independent of sex (Yohn et al., 2019), whereas 11-day SIS applied to adolescent male Balb/c mice did not affect risk-taking behavior when tested twenty days following SIS (de Lima and Massoco, 2017). Several articles report that SIS in mice did not affect (novelty-induced) locomotor activity in the OFT, and this was independent of SIS protocol duration or timing of the OFT (Dadomo et al., 2018; de Lima and Massoco, 2017; Scharf et al., 2013; Schmidt et al., 2010a; Sterlemann et al., 2008; Wang et al., 2019). However, when measured five weeks following SIS^49D^ in adolescent male CD1 mice, SIS vulnerable mice were hyperactive during the first three minutes of the OFT compared to SIS resilient mice and non-stressed controls (Schmidt et al., 2010b). Additionally, SIS^49D^ applied to adolescent male or female CD1 mice decreased (initial) locomotor activity compared to non-stressed controls when measured two months or roughly a week following SIS (Saavedra-Rodríguez and Feig, 2013; Schmidt et al., 2007). Remarkably, the hypoactivity during the OFT following an effect that was more pronounced in the F0 females than F0 males, was transmitted to the F1 offspring by mothers and fathers but only to F2 and F3 daughters by fathers (Saavedra-Rodríguez and Feig, 2013). SIS^49D^ applied to adolescent male or female C57BL/6J mice also decreased the distance travelled in the center zone of the open field arena in stressed male and female mice compared to non-stressed controls when measured several weeks after the SIS protocol, and this anxiogenic effect was dependent on the estrous cycle in female mice (Yohn et al., 2019). In contrast, SIS^49D^, albeit a slightly modified version with random cage distribution, applied to two large cohorts of adolescent male C57BL/6J mice consistently increased time spent in the center zone of the open field arena compared to non-stressed controls, when measured during the active (dark) phase five weeks following SIS (Sturman et al., 2021). During the novelty suppressed feeding test, SIS^49D^ applied to adolescent male CD1 mice or adolescent male and female C57BL/6J mice consistently increased latency to initiate food consumption (Schmidt et al., 2007, 2010a; Sterlemann et al., 2008; Yohn et al., 2019). This change in feeding latency was not observed one year following SIS^49D^ in adolescent male CD1 mice (Sterlemann et al., 2008).

#### 3.3.3 Cognition

Cognitive behavior in rodents is commonly assessed by tests that focus on learning and memory capabilities. For example, the Y-maze and MWM assess spatial learning and the retrieval of aversive emotional memories, whereas fear conditioning specifically measures contextual memory (Rudy et al., 2004). Other commonly used cognitive tests are the object recognition test and object location test.

In general, SIS negatively affects long-term (spatial) memory and learning capabilities in rats. Contextual memory, on the other hand, was only affected immediately following SIS and SIS did not produce prolonged memory impairments. A single study in mice assessing cognitive behavior indicates an impairment in hippocampus-dependent spatial memory (Sterlemann et al., 2010).

In adolescent male Long-Evans rats, SIS^16D^ improves within-day performance during acquisition learning in an MWM compared to non-stressed controls (Green and McCormick, 2013). However, between-day (*i*.*e*. between the first trial of the day and last trail of the previous day) performance during acquisition learning was decreased in stressed rats compared to non-stressed controls, indicating that SIS produces a modest impairment in long-term spatial memory, but not in short-term or working memory when tested six weeks following SIS (Green and McCormick, 2013). This impairment in long-term spatial memory was confirmed using the object recognition test or object location test in additional studies with adolescent male and female Long-Evans rats when tested immediately or four weeks following SIS (Green et al., 2013; McCormick et al., 2010, 2012). Additionally, SIS^16D^ applied to adolescent male Long-Evans rats decreases the latency to approach an object and the total time interacting with the object when tested six weeks following SIS (Green and McCormick, 2013). SIS also affects contextual short-term memory, as SIS^16D^ applied to adolescent male and female Long-Evans rats decreases freezing behavior during a fearful context in adolescent rats when tested immediately or four weeks following SIS (McCormick et al., 2013b; Morrissey et al., 2011). However, such a memory impairment was not observed when the SIS^16D^ protocol was applied during adulthood (Marcolin et al., 2020; Morrissey et al., 2011). Thirty-five-day SIS, applied either to adolescent or adult male Sprague-Dawley rats, produced more fear-potentiated startle behavior compared to non-stressed controls when tested immediately following SIS (Tsai et al., 2014). In contrast, 42-day SIS did not immediately affect acoustic startle behavior in adolescent male Wistar rats, with stressed rats even showing less startle behavior compared to non-stressed controls, when measured more than two months after SIS (Maslova et al., 2010).

SIS^49D^ applied to adolescent male CD1 mice impairs hippocampal-dependent spatial memory in the MWM and decreased exploration of a novel arm in the Y-maze compared to non-stressed controls when tested 12 months after SIS (Sterlemann et al., 2010).

#### 3.3.4 Social behavior

Since SIS particularly impacts the social dynamics of the experimental animals, all tests assessing social behavior are discussed in this section, even though some of these tests also measure anxiety-related aspects (social interaction test), depression-related aspects (sexual behavior) and memory-related aspects (social novelty test/social recognition test). Tests that assess aggression and maternal care have been included as well.

In general, both SIS^16D^ and SIS^49D^ decrease social interactions, an indication of anxiety-like behavior, in both rats and mice, respectively. Furthermore, SIS also negatively affects social memory in both species. SIS^16D^ impairs sexual behavior, a classic symptom of depression, in rats. Although most of the social behavioral tests were performed in rats, the studies that applied SIS to mice also generally observe impaired social behavior following SIS.

Three articles report that SIS^16D^ applied to adolescent male Long-Evans rats reduced social interaction time when tested immediately or four weeks following SIS (Green et al., 2013; Hodges et al., 2017, 2018). However, two studies that applied SIS^16D^ or 28-day SIS to adolescent male Long-Evans rats or adult female Sprague Dawley rats, respectively, did not observe significant changes in social interaction time following SIS when tested four or two weeks following SIS (Marcolin et al., 2020; Pittet et al., 2017). Twenty-eight-day SIS applied to adult female Sprague Dawley rats resulted in less aggression towards a social stimulus compared to controls (Pittet et al., 2017). Furthermore, 28-day SIS did not affect maternal aggression towards a male intruder, although stressed rats did groom their pups significantly more during the presence of the intruder (Pittet et al., 2017). Finally, the 28-day SIS did not affect general maternal care (*e*.*g*. grooming, licking and nursing) (Pittet et al., 2017). SIS^16D^ applied to adolescent male Long-Evans rats impaired sexual behavior throughout adulthood as stressed rats show less sexual behavior in general, had a longer latency to ejaculate and a lower total amount of ejaculations (McCormick et al., 2013a).

SIS^49D^ applied to adolescent male CD1 mice increased aggressive behavior compared to non-stressed controls (Schmidt et al., 2007). SIS^49D^ applied to adolescent male and female CD1 mice reduced direct social interactions with a same-sex juvenile compared to non-stressed controls when measured two months following SIS (Saavedra-Rodríguez and Feig, 2013). SIS^49D^ applied to adolescent male CD1 mice did not affect social interaction time with a male DBA mouse (Scharf et al., 2013) or the time spent in the chamber containing a same-sex stranger in the three-chambered social interaction test, in either male or female CD1 mice stressed during adolescence (Saavedra-Rodríguez and Feig, 2013). However, in the latter study, SIS^49D^ did decrease preference for social novelty (*i*.*e*. time spent in the chamber of a newly introduced stranger compared to the previously introduced stranger; Saavedra-Rodríguez and Feig, 2013).

### 3.4 Physiological impact of SIS

To assess the physiological response to SIS, several stress-related physiological parameters for stress have been reported, including stress hormone dynamics, changes in HPA-axis function, neurobiological aspects like neuronal morphology and hippocampal neurogenesis, and metabolic parameters. Sex hormone dynamics following SIS have been relatively understudied.

#### 3.4.1 Metabolic measurements

Commonly used parameters to evaluate the effect of SIS on energy metabolism, the HPA-axis or the cardiovascular system are changes in body weight, caloric intake, body fat composition, terminal adrenal or thymus weight, blood CORT levels, or blood pressure.

In general, SIS has either no effect or blunts body weight growth in both rats and mice. SIS decreases caloric intake, independent of the age of the stressed animals. Finally, SIS increases adrenal weight only in mice, and stressed mice generally show a worsened body fur condition.

SIS^16D^ applied to adolescent male and female Sprague Dawley rats or adolescent male and female Long-Evans rats blunted body weight growth in males, but not females, when measured immediately after SIS (Breach et al., 2019; McCormick et al., 2007). Thirty-five-day SIS applied to adolescent or adult male Sprague-Dawley rats blunted body weight growth in both males and females, independent of when SIS was applied, and these effects were associated with reduced caloric intake in all stressed experimental groups compared to non-stressed controls (Tsai et al., 2014). SIS^16D^ applied to adolescent male and female Sprague Dawley rats or 42-day SIS applied to male Wistar rats did not affect adrenal weight of stressed rats compared to non-stressed controls (Breach et al., 2019; Maslova et al., 2010). Forty-two-day SIS applied to male Wistar rats increased systolic arterial blood pressure when measured immediately after SIS, and this effect was more pronounced when measured again 70 days later (Maslova et al., 2010). One study assessed testosterone and observed that SIS^16D^ applied to adolescent male Long-Evans rats decreases testosterone levels at various timepoints following SIS compared to non-stressed controls (McCormick et al., 2013b).

In mice, the effects of SIS on body weight dynamics are inconsistent. Several articles report no effect of SIS^49D^ on body weight growth in adolescent male CD1 mice (Scharf et al., 2013; Schmidt et al., 2007, 2010b). However, two articles report decreased body weight growth either after SIS^49D^ applied to adolescent male CD1 mice (Boleij et al., 2014) or after 19-day SIS applied to young-adult male SJL mice (Chatterjee et al., 2009). The lag in body weight growth was normalized within two weeks following SIS^49D^ (Boleij et al., 2014). Twenty-eight-day SIS applied to adult female CD1 mice decreases body weight gain and reduces caloric intake compared to non-stressed controls (Dadomo et al., 2018). SIS^49D^ applied to adolescent male CD1 mice followed by twelve months of individual housing decreased subcutaneous white adipose tissue and visceral-to-subcutaneous white adipose tissue ratios, and these effects were prevented by paroxetine treatment during SIS^49D^ (Schmidt et al., 2009). Furthermore, SIS^49D^ applied to adolescent male CD1 mice generally increases adrenal weight and decreases thymus weight compared to non-stressed controls (Scharf et al., 2013; Schmidt et al., 2007, 2010a, 2010b; Sterlemann et al., 2008). SIS^49D^ applied to adolescent male CD1 mice also induces an impoverished fur condition, indicative of decreased general health in stressed mice compared to non-stressed controls (Boleij et al., 2014; Schmidt et al., 2007).

#### 3.4.2 HPA-axis function

CORT level dynamics, hypothalamic expression of *Nr3c1* [coding for the glucocorticoid receptor (GR) receptor] and expression of *Nr3c2* [coding for the mineralocorticoid receptor (MR) receptor] in various stress-related brain regions, and expression of *Avp* (coding for arginine vasopressin), *Crh* (coding for corticotropin-releasing hormone) and adrenocorticotropic hormone [ACTH; processed from *proopiomelanocortin* (POMC)] have all been reported as an indication of HPA-axis function following SIS.

In short, SIS severely impacts HPA-axis function in mice, independent of whether SIS^49D^ or 19-day SIS had been applied. Accordingly, SIS applied to mice affected gene expression of several stress behavior-related genes in the paraventricular nucleus (PVN) of the hypothalamus and the hippocampus. In rats, the data on SIS effects on HPA-axis function are inconsistent and minimal. This inconsistency appears independent of SIS protocol duration.

SIS^16D^ resulted in elevated, unaltered, and decreased plasma CORT levels when applied to adolescent male Long-Evans rats when measured at baseline, after fear conditioning, or after fear recall, respectively (Marcolin et al., 2020). SIS^16D^ did not alter plasma CORT levels when applied to adolescent male and female Long-Evans rats when measured after an acute stressor (McCormick et al., 2008; Roeckner et al., 2017), but did result in lower CORT levels when applied to adolescent male, but not female, Long-Evans rats when measured at the end of SIS (McCormick et al., 2007). Forty-two-day SIS applied to adolescent male Wistar rats did not affect plasma CORT levels when measured six weeks following SIS (Maslova et al., 2010), whereas 35-day SIS and 100-day SIS applied to adult male Wistar rats induced elevated plasma CORT levels compared to non-stressed controls (Eskandari Sedighi et al., 2015). SIS^16^^D^ applied to adolescent male Long-Evans rats increased *Crh* expression in the PVN compared to non-stressed controls (McCormick et al., 2007).

SIS^49D^ applied to adolescent male CD1 mice and 19-day SIS applied to young-adult male SJL mice increased plasma CORT levels in stressed mice compared to non-stressed controls (Chatterjee et al., 2009; Scharf et al., 2013; Schmidt et al., 2007, 2010a, 2010b; Sterlemann et al., 2008; Yohn et al., 2019). Most studies assessed plasma CORT levels immediately following the end of SIS. Following SIS^49D^ applied to adolescent male CD1 mice, plasma CORT elevations either returned to baseline after one week of individual housing (Schmidt et al., 2007) or stayed elevated after five weeks of individual housing (Scharf et al., 2013). The latter effect was absent following paroxetine treatment (Scharf et al., 2013). Circadian rhythms impact CORT dynamics, and SIS^49D^ applied to adolescent male CD1 mice increases CORT levels in stressed mice compared to non-stressed controls in the morning (Scharf et al., 2013; Sterlemann et al., 2008). In line with these circadian aspects, SIS^49D^ applied to adolescent male CD1 mice decreases plasma ACTH levels in the morning, whereas plasma ACTH was not affected in the evening (Schmidt et al., 2007). SIS^49D^ applied to adolescent male CD1 mice increases CORT/ACTH ratios, indicative of a dysregulated HPA-axis (Schmidt et al., 2007, 2010a). SIS^49^^D^ applied to adolescent male CD1 mice decreases or lowers *Crh* expression in the PVN, and these effects appear both immediate and long-lasting (Scharf et al., 2013; Schmidt et al., 2010a). SIS^49D^ applied to adolescent male CD1 mice also decreases *Nr3c1* and *Nr3c2* expression in the hippocampus, a brain region implicated in (spatial) memory (Scharf et al., 2013; Schmidt et al., 2007, 2010a; Sterlemann et al., 2008). Similar effects were observed in another study with adolescent male CD1 mice, but these effects appear dependent on whether mice had been tested in a behavioral assay before the assessment of gene expression (Boleij et al., 2014). Alterations in *Nr3c1* or *Nr3c2* expression were only present when measured directly after SIS^49D^, but when measured one month or one year after SIS, expression levels normalized again or even appeared increased (Scharf et al., 2013; Schmidt et al., 2010b; Sterlemann et al., 2008). SIS^49D^ applied to adolescent male CD1 mice increased *Avp* expression when measured immediately or twelve months following SIS (Scharf et al., 2013; Sterlemann et al., 2008).

#### 3.4.3 Neurobiological measurements

Several other readouts, including neuronal morphology, neural activation, neuroplasticity, and hippocampal neurogenesis, have been studied following SIS. Adult hippocampal neurogenesis is commonly assessed by quantifying Ki67 and BrdU, markers of cellular proliferation, and DCX, a marker for immature/new-born neurons (Lucassen et al., 2015).

In general, SIS affects neuronal morphology, activation and synaptic plasticity in rats. For mice, these parameters have unfortunately not been reported to date. With regards to hippocampal neurogenesis, SIS^16D^ in rats and SIS^49D^ in mice produce opposite effects on the total amount of immature neurons. It remains to be determined whether these opposite effects on hippocampal neurogenesis are species-specific, sex-specific, SIS protocol duration-dependent, or a combination of these factors.

SIS^16D^ applied to adolescent male and female Sprague Dawley rats reduced apical branch length and number in females and basal dendritic length and dendritic quantity in males compared to non-stressed controls (Breach et al., 2019). In addition, 35-day SIS applied to adolescent male Sprague Dawley rats decreased these parameters when applied during adolescence, but increased these parameters when applied during adulthood (Tsai et al., 2014). Furthermore, SIS^16D^ applied to adolescent male Long-Evans rats decreased neuronal activation (quantified as cFos-positive cells) in the PVN and arcuate nucleus of the hypothalamus, two brain regions implicated in stress behavior-related, compared to non-stressed controls when tested one day following SIS and following a brief social interaction test (Hodges et al., 2018). Despite these observations, no differences in neuronal activity (quantified as cFos-positive cells) were observed in brain areas related to social-behavior, including the medial amygdala, lateral septum, the CA2 subregion of the hippocampus, and the nucleus accumbens (Hodges et al., 2018). SIS^16D^ applied to adolescent male Long-Evans rats impacted hippocampal synaptic plasticity during adulthood, as expression of CaMKIIa and CaMKIIb, markers of synaptic plasticity, was increased in the dorsal hippocampus of stressed rats compared to non-stressed controls when tested four weeks following SIS (McCormick et al., 2012). In the same study, SIS^16D^ had minimal impact on hippocampal expression of T286a/b and Synaptophysin, additional marker of synaptic plasticity, compared to non-stressed controls (McCormick et al., 2012). The finding that SIS^16D^ impaired plasticity in the hippocampus, a region important for spatial memory, is expected given the earlier mentioned impairments in spatial memory (McCormick et al., 2012). SIS^16D^ applied to adolescent male Long-Evans rats did not impact synaptic plasticity in the nucleus accumbens and prefrontal cortex (as assessed using the markers Spinophilin, PSD95 and CaMKIIIa/b) when tested immediately following SIS (Marcolin et al., 2020). However, when tested four weeks following SIS, SIS^16D^ applied to adolescent male Long-Evans rats was associated with lower CaMKIIIa and PSD95, but not CaMKIIIb or Spinophilin, in the prefrontal cortex compared to non-stressed controls, without affecting these markers in the nucleus accumbens or dorsal or ventral hippocampus (Marcolin et al., 2020). Thirty-five-day SIS applied to adolescent or adult male Sprague Dawley rats decreased and increased, respectively, full-length brain-derived neurotrophic factor (BDNF) levels in the amygdala, a region known for processing emotions such as anxiety and aggression, compared to their respective controls when tested immediately following SIS (Tsai et al., 2014). Furthermore, the same study reports that truncated BDNF was increased in adult rats, SNAP-25 was decreased in adolescent rats, and Synaptogamin-1 was unaltered at both ages, compared to controls (Tsai et al., 2014). SIS negatively impacts neuronal development in the amygdala of the adolescent brain (as indicated by altered dendritic field and spine density of basolateral amygdala neurons), whereas amygdala neurons in adult rats seem more capable of adapting to SIS (Tsai et al., 2014). SIS^16D^ applied to adolescent male Long-Evans rats increased the number of hippocampal Ki67-positive cells when tested three days after the start of SIS (McCormick et al., 2012). However, when tested immediately or four weeks following SIS, hippocampal Ki67-positive cells were unaltered, whereas DCX-positive neurons were increased, compared to non-stressed controls (McCormick et al., 2012). SIS^16D^ applied to adolescent female Long-Evans rats decreased the number of hippocampal BrdU-positive cells compared to non-stressed controls when tested four days following SIS (McCormick et al., 2010). Furthermore, 100-day SIS applied to adult Wistar rats decreased cytoskeletal microtubular system in the brain (Eskandari Sedighi et al., 2015), which is essential for functions such as learning and memory (Bianchi et al., 2006), and neuronal plasticity (Bianchi et al., 2005). However, in the same study, these changes were not observed after 35-day SIS (Eskandari Sedighi et al., 2015).

SIS^49D^ applied to adult male and female C57BL/6J mice decreased the number of hippocampal Ki67-positive neurons in males and females, without hippocampal DCX-positive cells were only decreased in females when tested five weeks following SIS (Yohn et al., 2019). Nineteen-day SIS applied to young-adult male SJL mice induced complex changes in subunits of big potassium (BK) channels, which are involved in BK channel excitability, in the adrenal medulla and pituitary gland (Chatterjee et al., 2009).

## 4 Discussion

**Fig. 3:**
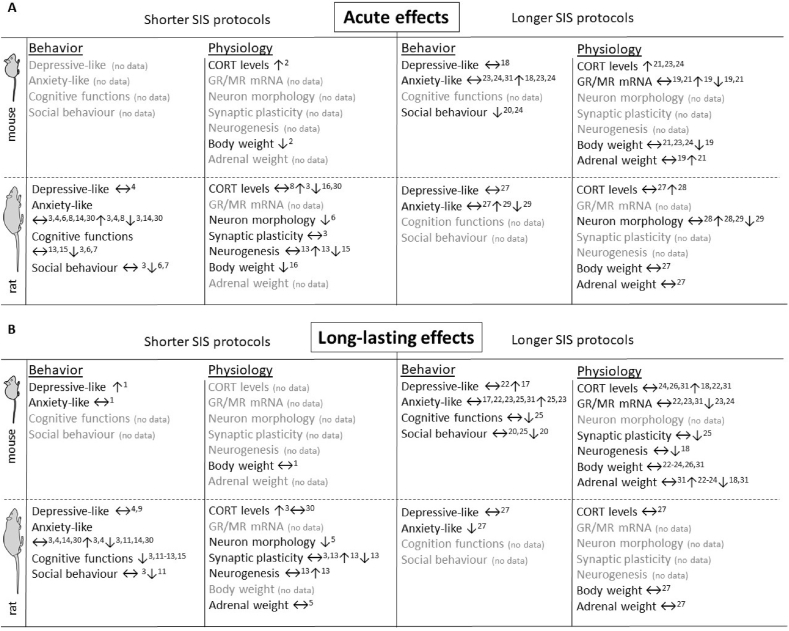
Immediate and long-lasting behavioral and physiological effects of relative short and long SIS protocols. Overview of (A) immediate behavioral and physiological effects (*i*.*e*. assessed within seven days after end of SIS protocol) and (B) long-lasting (*i*.*e*. assessed at eight days or later after end of SIS protocol) stress-related alterations after relative short (duration of 21 days or less) and relative long (duration of 35 days or longer) SIS protocols. Arrows indicate changes in SIS animals compared to non-stressed controls. ↔: similar to control. For details see Appendix A and B. ^1^de Lima and Massoco (2017) (11D, ♂); ^2^Chatterjee et al. (2009) (19D, ♂); ^3^Marcolin et al. (2020) (16D, ♂); ^4^Marcolin et al. (2019) (16D, ♂); ^5^Breach et al. (2019) (16D, ♂&♀); ^6^Hodges et al. (2018) (16D, ♂); ^7^Hodges et al. (2017) (16D, ♂); ^8^Roeckner et al. (2017) (16D, ♂&♀); ^9^McCormick et al. (2013a) (16D, ♂); ^10^McCormick et al. (2013b) (16D, ♀); ^11^Green et al. (2013) (16D, ♂); ^12^Green and McCormick (2013) (16D, ♂&♀); ^13^McCormick et al. (2012) (16D, ♂), ^14^Morrissey et al. (2011) (16D, ♂); ^15^McCormick et al. (2010) (16D, ♀); ^16^McCormick et al. (2007) (16D, ♂&♀); ^17^Wang et al. (2019) (49D, ♂); ^18^Yohn et al. (2019) (49D, ♂&♀); ^19^Boleij et al. (2014) (49D, ♂); ^20^Saavedra-Rodríguez and Feig (2013) (49D, ♂&♀); ^21^Schmidt et al. (2010a) (49D, ♀); ^22^Schmidt et al. (2010b) (49D, ♂); ^23^Sterlemann et al., (2008) (49D, ♂); ^24^Schmidt et al. (2007) (49D, ♂); ^**25**^Sterlemann et al. (2010) (49D, ♂); ^26^Schmidt et al. (2009) (49D, ♂); ^27^Maslova et al. (2010) (42D, ♂); ^2^^8^Eskandari Sedighi et al. (2015) (35D, ♂); ^29^Tsai et al. (2014) (35D, ♂); ^30^McCormick et al. (2008) (16D, ♂&♀); ^31^Scharf et al. (2013) (49D, ♂).

Both relative long and short SIS protocols in mouse and rat induce a variety of stress-related behavioral and physiological parameters (see Fig. 3). This systematic review evaluated 33 articles, of which the majority applies the SIS^16D^ or SIS^49D^ protocol. To date, SIS^16D^ has only been applied to rats, whereas SIS^49D^ has only been applied to mice. Nonetheless, several articles reported the impact of relative short SIS protocols (*e*.*g*. 11 or 19 days) in mice (Chatterjee et al., 2009; de Lima and Massoco, 2017) or relative long SIS protocols (*e*.*g*. 35 or 42 days) in rats (Eskandari Sedighi et al., 2015; Maslova et al., 2010). Despite severely limited in number, these studies do facilitate a careful comparison between the effectiveness of relative short and long SIS protocols in mice or rats.

### 4.1 Duration-specific effects

Both relative short and long SIS rat protocols altered anxiety-like behavior, increased CORT levels, altered neuron morphology and generally blunted body weight gain (see Appendix A), suggesting comparable effectiveness. However, the three articles that applied relative long SIS (Eskandari Sedighi et al., 2015; Maslova et al., 2010; Tsai et al., 2014), unfortunately assessed a limited number of parameters compared to the articles with relative short SIS protocols. For example, these three studies did not assess the effect of SIS on cognitive behavior, social behavior, synaptic plasticity or neurogenesis. Furthermore, a relative short SIS protocol decreased body weight gain in stressed rats compared to non-stressed controls (Breach et al., 2019), whereas a relative long SIS protocol did not affect this parameter (Maslova et al., 2010).

Both relative short and long SIS mouse protocols increase depressive-like behavior, elevate CORT levels and generally decrease body weight gain (see Appendix B), suggesting comparable effectiveness. However, the two articles that applied a relative short SIS mouse protocol (Chatterjee et al., 2009; de Lima and Massoco, 2017) unfortunately assessed a limited number of parameters compared to the relative long protocol studies, and did not assess the impact of SIS on social behavior, gene expression, neurogenesis or adrenal weight. Furthermore, relative long SIS protocols increase anxiety-like behavior (Schmidt et al., 2007; Sterlemann et al., 2008, 2010; Yohn et al., 2019) or decrease anxiety-like behavior (Sturman et al., 2021) in SIS mice compared to non-stressed controls, whereas a relative short SIS protocol did not affect this parameter (de Lima and Massoco, 2017).

Thus, although it seems that relative long SIS protocols are more effective compared to relative short protocols in mice, and relative short SIS protocols seem more effective compared to relative long protocols in rats, the underlying studies vary in methodological design to such an extent that results are often incomparable. Variation in methodological design includes differences in species, strain, protocol length, age, cage density, cage change frequency, isolation between cage change, and (timing of) molecular/behavioral readout. We thus echo the sentiment that establishment of a standardized SIS protocol would increase reproducibility and thus utility of this etiologically relevant stress model (Goñi-Balentziaga et al., 2018; Lopez and Bagot, 2021). Due to the limited number of articles with relative short SIS protocols in mice and relative long SIS protocols in rats, a conclusive comparison of different protocol duration effectiveness is currently not possible without having species-specific differences as a confounding factor (see Fig. 3).

### 4.2 Species-specific effects of SIS

In both mouse and rat, SIS increases plasma CORT levels and generally decreases body weight gain, irrespective of protocol duration (see Appendix A and B). Furthermore, the effects of SIS on social behavior were rather similar in mice and rats (Green et al., 2013; Hodges et al., 2017, 2018; Marcolin et al., 2020; Saavedra-Rodríguez and Feig, 2013; Schmidt et al., 2007), even though the natural social structure in these species is very different (Blanchard et al., 2001; Ellenbroek and Youn, 2016). Notably, SIS protocols induced depressive-like behavior in mice (de Lima and Massoco, 2017; Wang et al., 2019), but this was not observed in rats (Marcolin et al., 2019; Maslova et al., 2010; McCormick et al., 2013b). Several studies consistently report that SIS deteriorates cognitive capacities in rats (Green et al., 2013; Green and McCormick, 2013; Hodges et al., 2017, 2018; Marcolin et al., 2020; McCormick et al., 2010, 2012), whereas only one study suggests a cognitive impairment in mice (Sterlemann et al., 2010). To date, neuron morphology has only been investigated in rats (Breach et al., 2019; Eskandari Sedighi et al., 2015; Hodges et al., 2018; Marcolin et al., 2020; McCormick et al., 2012; Tsai et al., 2014), whereas gene expression has only been investigated in mice (Boleij et al., 2014; Scharf et al., 2013; Schmidt et al., 2007, 2010a, 2010b; Sterlemann et al., 2008). As several parameters have not been consistently investigated in both species, and because relative short SIS protocols are generally applied to rat and relative long SIS protocols to mouse, a comprehensive comparison of the species-specific effects of SIS is currently not possible. Additional studies are needed to fill this literature gap and enable a proper comparison between different SIS protocol durations in mouse and rat.

Based on the current literature that SIS induces a wide range of stress-related behavioral and physiological impairments in mouse and rat, we conclude that SIS is effective in inducing social stress in both species. It remains unclear whether relative long SIS (*e*.*g*. SIS^49D^) is a more effective protocol than relative short protocols, or whether mice are generally more susceptible to the effects of SIS.

### 4.3 Density-specific effects of SIS

Differing variation in cage density is another important factor between SIS protocols that contributes to the variable findings observed following various SIS protocols. During SIS^16D^, each cage contains two rats, whereas during SIS^49D^, each cage contains four mice. The social density of a cage can affect several stress-related measures, as studies indicate that greater cage density is associated with elevated plasma CORT and increased adrenal weight (Laber et al., 2008; Paigen et al., 2012). However, several SIS protocols with different cage densities (*e*.*g*. 5 rats/cage or 2 rats/cage) induce similar behavioral and physiological stress-related alterations (Breach et al., 2019; de Lima and Massoco, 2017; Tsai et al., 2014; Wang et al., 2019). In addition, SIS protocols with similar cage density, but different experiment duration and/or species, had differential effects on anxiety-like behavior (de Lima and Massoco, 2017; Roeckner et al., 2017; Schmidt et al., 2010a; Sterlemann et al., 2008), suggesting that protocol duration might a dominant factor over cage density. Furthermore, a 35-day SIS protocol in rat, housing ten animals per cage, produced minimal behavioral and physiological alterations (Maslova et al., 2010). Thus, it is possible that this number of animals per cage functions as a social buffer, as previous research has shown that social presence and/or physical contact can reduce stress-related physiological effects (Morrison, 2016), therefore potentially diminishing the negative effects of SIS.

Taken together, these findings indicate that cage density is likely not a primary factor that directly impacts effectiveness of relative short and long SIS protocols, but a potential social buffering effect of high cage densities cannot be excluded.

### 4.4 Sex-specific effects of SIS

Apart from species-specific differences, it has been debated whether the SIS paradigm is effective in both sexes (Haller et al., 1999; Palanza, 2001; Roeckner et al., 2017). Several articles advocate that SIS can be optimally applied to female rodents, as females are more susceptible to the consequences of social stress (Haller et al., 1999; Palanza, 2001). This is supported by evidence suggesting different coping mechanisms between sexes, as females are more likely to "tend and befriend” in stressful situations, whereas males rather "fight or flight” (Taylor et al., 2000; also see Box 1). These sex-specific differences become apparent when assessing behavior during stressful tasks (Archer, 1975).

BOX 1 **The role of aggression during SIS**

The recurring formation of new social hierarchies, a major component of the unpredictable chronic stress during the SIS paradigm, is often associated with aggressive behavior, especially in male mice. However, the duration and intensity of aggression within an experimental cohort can significantly impact the development of physiological and behavioral adaptations, thus affecting reproducibility of the SIS paradigm within and between laboratories. Here we provide several tips for optimal modulation, monitoring and scoring of aggression during SIS protocols.

**Modulation of aggression through experimental design**

Several factors can modulate aggressive behavior within a cohort and limit physical injuries on an individual level. When designing a SIS experiment, we recommend considering the following factors to restrict excessive aggression levels:

- Provision of cage enrichment, especially nesting material, and adequate cage floor area.
- Unrestricted access to food and water.
- Absence of the opposite sex in the same experimental room, especially with male rodents.
- Proper selection of the rodent strain, potentially avoiding high-aggression strains.
- Removal of the uninjured animal when multiple cagemates have injuries (the injury-free animal is likely the aggressive and biting conspecific).
- When aggression levels are generally high on a cohort level, and the choice of rodent strain cannot be altered, clipping of teeth can limit the number of injuries.

**Monitoring and scoring of aggression during an experiment**

For optimal reporting of SIS-induced behavioral and neurobiological (mal)adaptations and to increase reproducibility of the SIS paradigm, aggressive behavior should be monitored and scored adequately. We recommend doing the following, preferentially at least weekly:

- Assessment of fur state of each experimental animal (also see Mineur et al., 2003).
- Assessment of amount and location of injuries (also see Alleva, 1993).
- When available, video monitoring can be used for detailed assessment of individual aggressive behavior (also see Brain et al., 1981).
- During observation of (continued) excessive aggression, remove aggressor from cohort as soon as possible.
- When a humane endpoint is reached, remove experimental animal as soon as possible and follow institutional ethical procedures.

Alt-text: BOX 1

One article also advocates that SIS is less suitable for studies in females because SIS does not induce stress-related behavior, such as altered anxiety-like behavior or ethanol preference, in females (Roeckner et al., 2017). However, this article reported that stressed male and female rats exhibit similar behavioral impairments in the EPM, had equal total ethanol intake, and similar changes in plasma CORT levels (Roeckner et al., 2017). Additionally, several studies have shown similar SIS-induced behavioral alterations in males and females (McCormick et al., 2013b; Morrissey et al., 2011; Yohn et al., 2019), suggesting applicability of SIS to both sexes. Nonetheless, several studies report differential effects of SIS in male and female rodents. For example, SIS^16D^ decreased body weight growth and plasma CORT levels in male rats, whereas SIS^16D^ did not alter these parameters in females (McCormick et al., 2007, 2008). Neuronal morphology is negatively affected in both stressed male and female rats, but in a sex-specific manner (Breach et al., 2019).

Taken together, the data indicate that SIS is effective in both sexes, although sex-specific changes in specific behavioral or physiological stress-related parameters can be observed.

### 4.5 Immediate versus long-lasting effects of SIS

Eleven studies investigate the immediate impact of SIS (assessed within seven days; Fig. 3A). Eleven studies investigate the long-lasting effect of SIS (assessed eight days or later; Fig. 3B). Eleven studies investigate both the immediate and long-lasting effect of SIS (see Fig. 3A and B). Thirty-five days after the end of the SIS^49D^ protocol, plasma CORT levels in one cohort of stressed mice remained elevated compared to non-stressed controls (Schmidt et al., 2010b), whereas the same laboratory had previously reported that the increases in plasma CORT levels returned to baseline within a week (Schmidt et al., 2007). Such differential observations can potentially be explained by individual differences to SIS susceptibility (see *Interindividual differences of SIS* section below), especially since the used mouse cohorts were from the CD1 outbred strains and this strain might have higher variability due to genetic predispositions than inbred strains. It could also be explained by differing general aggression levels between both SIS cohorts (also see *Strengths and limitations* section below).

Three mouse studies have investigated both the immediate and long-lasting effects of SIS^49D^ on behavioral and physiological parameters in the same cohort, allowing direct comparison (Scharf et al., 2013; Sterlemann et al., 2008; Wang et al., 2019). Several physiological alterations that were observed immediately after the SIS protocol, such as increased adrenal weight and plasma CORT levels, were not observed when measured twelve months (during which mice were individually housed) after SIS^49D^ (Sterlemann et al., 2008) or were even decreased with regards to adrenal weight (Scharf et al., 2013). Similarly, the majority of behavioral impairments failed to persist (Wang et al., 2019). Notably, a decrease in number of head dips in OFT was observed one year following SIS^49D^, whereas this effect was not observed when tested immediately after the SIS protocol (Sterlemann et al., 2008). Two studies investigated the long-lasting effects of the SIS^49D^ protocol by only assessing behavioral and physiological parameters twelve months after the SIS protocol (Schmidt et al., 2009; Sterlemann et al., 2010).

Other studies have also reported a delayed onset of behavioral alterations. Initially, explorative behavior is normal following a rat SIS^16D^ protocol, whereas stressed rats spent less total time investigating a novel object 25 days after the SIS protocol compared to non-stressed controls (McCormick et al., 2010, 2012). In addition, a delayed onset of a physiological change has also been reported, as a 42-day rat SIS protocol increased systolic arterial blood pressure 70 days after the SIS protocol (Maslova et al., 2010). In mice, the negative effects of SIS on *Crh* expression in the PVN are amplified after twelve months of individual housing (Scharf et al., 2013).

Taken together, these findings emphasize the importance of consistent timing of behavioral and physiological assessment. It is possible that behavioral and/or physiological alterations are sometimes missed because they develop after tests have been performed. Conclusiveness could be achieved by measuring physiological and behavioral parameters at multiple timepoints following the SIS protocol (*e*.*g*. also six weeks later). Conversely, the examples above illustrate that not all direct behavioral and physiological changes are long-lasting and that many physiological and/or behavioral impairments ameliorate or even normalize over time.

### 4.6 Age-specific effects of SIS

Social interactions during early life and adolescence are important for the formation of normal social behavior (Spear, 2000). During this important developmental phase, social play behavior peaks in both humans and rodents and more time is spent on social interaction relative to other ages (Spear, 2000). Likewise, the consequences of mental health disorders, such as depression, during adolescence are severe and long-lasting as relapse rates are approximately 60% (Birmaher et al., 2002; Ginsburg et al., 2014). Adolescents have a general higher risk to develop mental health disorders and this is dependent on their perception of social stress levels (Mason et al., 2016). Coping mechanisms for stress also develop during adolescence (Sachser et al., 2011). Therefore, this vulnerable age group may particularly benefit from mechanistic insight of studies conducting SIS protocols during adolescence, as this paradigm was created in order to investigate the effects of chronic stress exposure during adolescence. In line with this, the age of experimental animals during SIS (adolescent versus adult) is an important factor when interpreting the efficiency of SIS to induce stress-related (mal)adaptations. Thus, future studies hold great promise to investigate the underlying mechanisms in the development of mental health disorders during adolescence, and the protective potential of behavioral (*e*.*g*. exercise training) or pharmacological interventions.

### 4.7 Interindividual differences of SIS

One mouse study explored the role of individual vulnerability to SIS^49D^ (Schmidt et al., 2010b). In this study, stressed mice were divided into three different groups based on their plasma CORT levels (*i*.*e*. top 20%, lowest 20%, and all remaining mice) five weeks after the SIS protocol (Schmidt et al., 2010b). The lowest CORT level group was considered ‘resilient’ whereas the highest CORT level group was considered ‘vulnerable’ to SIS (Schmidt et al., 2010b). Vulnerable mice had significantly larger adrenals, and higher *Nr3c2* expression levels in the hippocampus (Schmidt et al., 2010b). Initially, no group differences in behavioral parameters were seen between SIS mice and non-stressed controls (Schmidt et al., 2010b). However, when the groups were assessed separately, vulnerable animals exhibited more anxiety- and depressive-like behavior compared to resilient mice and non-stressed controls (Schmidt et al., 2010b). Aside from experimental timing, other studies may have failed to observe significant behavioral impairments simply because they averaged all stressed animals during analysis.

A recent article reported two identical SIS^16D^ cohorts with male Long-Evans rats (Marcolin et al., 2020). In the first cohort, SIS did not affect ethanol consumption, but in the second cohort SIS decreased ethanol consumption compared to non-stressed controls (Marcolin et al., 2020). The authors suggest that the different observations in these two cohorts results from variability in susceptibility to the SIS protocol (Marcolin et al., 2020). As previously mentioned, it is likely that some variation can be assigned to individual differences in stress susceptibility. However, the substantial size of both cohorts (*i*.*e*. 96 and 100 rats respectively), make it unlikely that these differences are solely the consequence of individual differences in SIS susceptibility.

### 4.8 Trans-generational effects of SIS

In mice, SIS can also negatively impact health in next generations. Unstressed female offspring of SIS^49D^ parents exhibit increased anxiety-like behavior, decreased social behavior, and elevated plasma CORT levels compared to unstressed control offspring from unstressed parents (Saavedra-Rodríguez and Feig, 2013). This study also performed cross-fostering experiments and revealed that the transmission is genetic and not the consequence of impaired postnatal nursing. The stress-induced behavioral and physiological alterations were transferred when a single parent was exposed to SIS^49D^, but the trans-generational effects of SIS were strongest when both the mother and father had experiences SIS. Thus, parental SIS alters the behavior and physiology of (unstressed) offspring, and these effects were apparent for several generations (Saavedra-Rodríguez and Feig, 2013). As this study is currently the only study reporting transgenerational effects of SIS in rodents, replication of these findings is important to further evaluate the processes underlying these (mal)adaptations in offspring.

### 4.9 Strengths and limitations

This review focuses on the effect of SIS protocols on stress-related behavioral and physiological parameters. The exclusion of studies that directly combined SIS and another stressor (*e*.*g*. early-life stress or predator exposure) or that did not include a SIS-only control group (see Supplemental Table 1), allowed an attempt to compare the effectiveness of various SIS protocols. However, most articles used the SIS^16D^ or SIS^49D^ protocol and these protocols have been applied specifically to either rat or mouse, respectively. Due to this limited application of relative short SIS protocols in rats and relative long SIS protocols in mice, findings from relative short SIS protocols can be minimally translated to relative long SIS protocols and *vice versa*. Furthermore, not all studies adequately report at what exact age the behavioral and physiological tests were performed or when the rodents were sacrificed. Including such important information, for example by reporting studies according to the ARRIVE guidelines, would cover the essential requirements for proper reproducibility and enable better comparisons (Percie du Sert et al., 2020). Altogether, this review provides a comparative overview of the current SIS protocol literature in rat and mouse, identifies remaining gaps in the literature, and provide practical recommendations to optimize reproducibility of SIS protocols.

SIS is an etiologically valid paradigm to model social stress-induced impairments in humans. First, SIS has construct validity as social stress is an important factor in the development of stress-related disorders in humans (Van Praag, 2004). Second, SIS has face validity as it induces emotional (anhedonia), metabolic (impairments in body weight growth and caloric intake) and psychomotor (increased anxiety-like behavior) symptoms (McCormick and Green, 2013; Schmidt et al., 2008). Third, SIS has predictive validity as treatment with anxiolytic (buspirone) or antidepressant (paroxetine/fluoxetine) drugs during or after SIS ameliorates SIS-induced anxiety- and depressive-like behaviors (Haller et al., 2004; Scharf et al., 2013; Schmidt et al., 2009; Yohn et al., 2019).

SIS protocols also have several advantages over other (chronic) stress models. SIS protocols can be easily and successfully applied in males and females. In addition, the recurring changes in home-cage hierarchy continue to produce unpredictable social stress, which should maintain sensitivity to the social stress. SIS protocols are also easy to upscale, making it potentially high-throughput, and are relatively low in labor intensity. A SIS protocol works with a minimal cage density of two, but cage density can be increased to upscale the number of experimental animals. One should however note that the number of cage mates, as well as cage area size, will likely impact the aggressiveness of the animals and will thus impact the intensity of the social stress. A SIS protocol can last multiple weeks to months, making it a bonafide chronic stress model. Because of the easiness to apply SIS protocols for a longer duration, social stress can be applied to adolescent animals and last into adulthood. This allows for the possibility to study the effects of social stress spanning multiple ages. When started during adulthood compared to adolescence, SIS is often associated with more aggression. Males also demonstrate more aggression during SIS, especially during adulthood, compared to females. Although one should always carefully monitor the health of each individual experimental animal, this is especially true for SIS cohorts of (adult) males.

On a practical level, SIS protocols also have several disadvantages. Although it is easy to upscale a SIS protocol, a substantial number of animals is minimally needed to ensure that experimental animals are always forming a new hierarchy with (an) unfamiliar cage mate(s). This large minimal number of animals also makes it less applicable to study the influence of genetic factors (*e*.*g*. in a transgenic line) on SIS susceptibility, as it requires substantial breeding cohorts. The very large number of animals needed for a SIS protocol also makes it challenging to test males and females at the same time, and to directly compare sex-specific effects. The chronic nature of longer SIS protocols, and the possibility to study the effects of social stress spanning multiple ages, can also be a disadvantage as it limits the possibility to study the deleterious effects of SIS during a specific age. Finally, the presence of one or multiple females in the home-cage or in the housing room will increase aggression levels in males, and separate rooms for male and female cohorts are thus recommended.

### 4.10 Future directions

Additional studies are required to better understand the immediate and long-lasting stress-related impact of SIS, and these studies should consider the contribution of protocol duration, species, developmental stage, interindividual differences and cage density. Establishment of a standardized SIS protocol would increase reproducibility and thus utility of this etiologically relevant stress model (also see Box 2). A direct comparison between rat and mouse, preferably with such a standardized protocol, can potentially reveal species-dependent effects of SIS, whereas a direct comparison between relative short and long protocols, in either rat or mouse, can potentially reveal duration-dependent effects of SIS. Such comparative studies would be very informative. Furthermore, we recommend considering interindividual differences in susceptibility to SIS by distinguishing between SIS resilient and susceptible animals using compiled assessment from multiple tests, preferably that depend on different behavioral or emotional states (*e*.*g*. motor versus affective), into a Z score (Ritov et al., 2016). In addition, it would be very informative to identify dominant and subordinate animals in SIS cohorts, and their individual susceptibility to SIS, as previous research suggests that social hierarchical status is a predicator of susceptibility to stress (Larrieu and Sandi, 2018).

BOX 2 **Practical recommendations on the design and reporting of a SIS experiment**

Proper study design as well as detailed reporting of the used methods are essential for utility and reproducibility of protocols and optimal applicability of the SIS paradigm to stress-related preclinical research. Here we list recommendations to standardize SIS protocols and to optimize the reporting of SIS protocols and related findings.

**Design of a SIS experiment**

• Consider experimental design factors that modulate aggression (see Box 1).
• Preferentially apply the commonly used SIS^16D^ or SIS^49D^ protocol. Deviation in duration and cage density will impair comparison between studies and labs.
• Design and use a randomization schedule to prevent housing with familiar conspecifics.
• Use a robust identification system (*e.g.* ear tags).
• Preferentially clean cages and alter cage composition at consistent intervals and time of day (*e.g.* onset dark phase) to increase reproducibility between labs.
• When possible, collect blood during and/or after SIS, and tissue (*e.g.* adrenals, thymus) after SIS to identify adaptations in stress-related physiology.
• Apply correct application of statistical analysis, including multiple testing correction, when applicable.

**Reporting of a SIS experiment**

The following aspects should be reported to optimize reproducibility of SIS protocols:

• Animals
  Rodent strain; distributor; sex; age upon arrival and/or start of SIS; total number of animals used (experimental animals versus controls); total number of animals removed due to humane endpoint; total number of hyperaggressive animals removed from cohort; type of animal identification used; and if teeth clipping was applied.
• SIS protocol
  Duration acclimatization animal facility; cage density during acclimatization; duration of SIS protocol; cage density; frequency and timing of cage composition alteration; time until behavioral/physiological assessment; randomization schedule used; long-term housing condition (*e*.*g*. five animals/cage) and duration following SIS.
• Environment
  Light schedule; lights on; cage size and type (*e*.*g*. open or IVC); type of bedding and cage enrichment; diet; frequency and timing of cage cleaning; presence additional animals (especially other sex); housing conditions until behavioral/physiological assessment.
• Health indicators
  Evolution of body weight, fur state, and amount and location of injuries per animal.
• Behavioral or physiological assessment
  Exact (time of) day when test/assessment is performed; lighting condition (light/dark) and intensity (lux) during test; housing condition (*e*.*g*. 24h isolation)) and duration before behavioral testing or physiological analysis; timing and method of sacrifice; statistics applied, including multiple testing correction, and used software; effect size and/or confidence interval; individual data points; number of animals removed from data set (including the reason).

Alt-text: BOX 2

### 4.11 Conclusion

In summary, both relative short and long SIS protocols induce a wide range of stress-related behavioral and (neuro)biological (mal)adaptations. Due to the varying quality and depth of current SIS articles in rat and mouse, and the variability in results therein, it is currently not possible to establish a protocol preference, irrespective of species. While mice in general may be more susceptible to social stress than rats, it is also possible that a longer exposure is minimally required for most behavioral and physiological stress-related alterations in mouse. Establishment of a standardized SIS protocol is important to increase utility of this etiologically relevant stress model (also see Box 2). Comprehensive analysis of the behavioral and physiological (mal)adaptations following such a standardized protocol, and the contribution of interindividual differences (including social rank), will provide valuable insight into the role of (chronic) social stress in the development and pathophysiology of depressive- and anxiety disorders.

## Author contributions

Amber Koert: Conceptualization, Methodology, Investigation, Writing – Original Draft, Writing – Reviewing & Editing; Annemie Ploeger: conceptualization, supervision, Writing – Reviewing & Editing; Claudi L.H. Bockting: supervision; Mathias V. Schmidt: Writing – Reviewing & Editing; Paul J. Lucassen: Supervision, Writing – Reviewing & Editing; Anouk Schrantee: Funding acquisition, Conceptualization, Supervision, Writing – Reviewing & Editing; Joram D. Mul: Funding acquisition, Conceptualization, Supervision, Writing – Reviewing & Editing.

## Funding sources

This work was supported by a grant from the 10.13039/501100001827Centre for Urban Mental Health, University of Amsterdam.

## Declarations of competing interest

None.

## Figures and Tables

**Table:** 

Article	Sample	SIS protocol	Acute/Delayed	Behavioral results	Physiological results
**Marcolin et al. (2020)**	• Long-Evans rats ♂ (Charles-River) • N = 192 (96 SIS; 96 CON)	• SIS^16D^: Pair (2/cage) changed daily for 16 days after 1 h isolation • P30–P45 (adolescence)	• Start behavioral tests: P47–P66 (ethanol test) & P70–P76 (C2 only) • Brain collection: P46 & P70 • Blood sample: P70–P76	• Chronic intermittent drinking model: (ethanol intake C1): SIS = CON (ethanol intake C2): SIS < CON • Ethanol intake over 24h (after withdrawal) SIS < CON • EPM: (open arms): SIS = CON (center): SIS > CON (closed arms): SIS < CON (head dips): SIS > CON • Social interaction (time): SIS = CON • CFC (freezing behavior): SIS = CON	• Plasma CORT: SIS > CON • Synaptic plasticity (Spinophilin, PSD95, CaMKIII): (acute, delayed) SIS = CON

**Marcolin et al. (2019)**	• Long-Evans rats ♂ (Charles-River) • N = 136 (68 SIS; 68 CON)	• SIS^16D^: Pair (2/cage) changed daily for 16 days after 1 h isolation • P30-45 (adolescence)	• Start behavioral tests: P47–P57 & P71–P81	• Ethanol intake: (acute) SIS > CON; (delayed) SIS = CON • SPT: (sucrose preference): (acute/delayed) SIS = CON (sucrose preference with competition): (acute/delayed) SIS > CON	

**Breach et al. (2019)**	• Sprague-Dawley rats ♂ & ♀ (Envigo) • N = 24 (12 ♂: 6 SIS; 6 CON, 12 ♀: 6 SIS; 6 CON)	• SIS^16D^: Pair (2/cage) changed daily for 16 days after 1 h isolation • P30–P45 (adolescence)	• Brain/organ collection: P79 • Body weight: P70–P79 • Estrous phase characterization (vaginal smears): P79		• Body weight gain: SIS ♂< CON ♂; SIS ♀ < CON ♀ • Adrenal glands: SIS ♂ = CON ♂; SIS ♀ = CON ♀ • Dendritic spine density mPFC: (apical branch nr): SIS ♂ = CON ♂; SIS ♀ < CON ♀ (apical branch length): SIS ♂ = CON ♂; SIS ♀ < CON ♀ (apical branch length): SIS ♂ = CON ♂; SIS ♀ < CON ♀ (branch per basilar tree): SIS ♂ < CON ♂; SIS ♀ = CON ♀ (length basilar tree): SIS ♂ < CON ♂; SIS ♀ = CON ♀ (thin spine density): SIS ♂ < CON ♂; SIS ♀ < CON ♀

**Hodges et al. (2018)**	• Long-Evans rats ♂ (Charles-River) • N = 62 (34 SIS; 28 CON)	• SIS^16D^: Pair (2/cage) changed daily for 16 days after 1 h isolation • P30–P45 (adolescence)	• Start behavioral tests: P46–P47 • Brain collection: P46	• Social interaction (time): SIS < CON • EPM: (open arms): SIS = CON (locomotor activity): SIS = CON	• Neural activation: (Fos-ir in PVN): SIS < CON (Fos-ir in ARC): SIS < CON (Fos-ir in NaC/Amygdala/dLS/CA2): SIS = CON

**Hodges et al. (2017)**	• Long-Evans rats ♂ (Charles-River) • N = 171 (83 SIS; 88 CON)	• SIS^16D^: Pair (2/cage) changed daily for 16 days after 1 h isolation • P30–P45 (adolescence)	• Start behavioral tests: P45/P46 • Brain collection: P46	• Social interaction (time): SIS < CON • Social recognition test (investigating): SIS < CON • CPP (conditioned chambers): SIS < CON	• Oxytocin receptor (in dLS & Nac Shell): SIS > CON • Vasopressin 1A receptor: SIS = CON

**Pittet et al. (2017)**	• Sprague Dawley rats ♀ (Charles-River) • N = 90 (60 ♀: SIS; 30 CON, 30 males)	• Other: Group composition (3/cage) changed every 2/3 days for 28 days • P74-102 & P84-112 (adulthood)	• Start behavioral tests: P109 & P119	• Saccharin preference: SIS = CON • Social interaction: (proximal to stimulus animal) SIS = CON (olfactory investigation) SIS = CON (aggression) SIS < CON • Predatory behavior: (latency first contact) SIS = CON (latency to attack) SIS > CON • Maternal care: (grooming/licking/nursing) SIS = CON • Maternal aggression test: (aggression towards male intruder) SIS = CON (grooming after intruder) SIS > CON	

Roeckner et al. (2017)	• Long-Evans rats ♂ & ♀ (unknown) • N = 56 (24 ♂: 6 SIS; 6 CON, 12 other 32 ♀: 8 SIS; 8 CON, 16 other	• SIS^16D^: Pair (2/cage) changed daily for 16 days after 1 h isolation • P30–P45 (adolescence)	• Start behavioral test: P49–P50 • Ethanol drinking paradigm:males P55–P81, females 57–P83 • Blood sample: P51	• EPM (open arms) SIS ♂ = CON ♂; SIS ♀ = CON ♀ • Ethanol preference: (ethanol intake): SIS ♂ = CON ♂; SIS ♀ = CON ♀ (ethanol preference): SIS ♂ > CON ♂; SIS ♀ = CON ♀	• Plasma CORT: SIS ♂ = CON ♂; SIS ♀ = CON ♀

**Eskandari Sedighi et al. (2015)**	• Wistar rats ♂ (unknown) • N = 18 (12 SIS; 6 CON)	• Other: Group (6/cage) composition changed 3 times weekly (by switching 2 random animals between groups) for 35 or 100 days • ±P105–P140 & ±P105–P205 (adulthood)	• Blood sample: P140 (35 day group) P205 (100 day group) • Brain collection: P140 (35 day group) P205 (100 day group)		• Microtubules: (GTPase activity): (35days) SIS = CON; (100days) SIS < CON (polymerization): (35days) SIS = CON; (100days) SIS < CON (dynamicity): (35days) SIS = CON; (100days) SIS < CON • Plasma CORT: (35/100days) SIS > CON

**Tsai et al. (2014)**	• Sprague-Dawley rats ♂ (Cheng Kung University) • N = 70 (36 SIS; 34 CON)	• Other: Group (5/cage) composition changed daily for 35 days after 1h isolation • P28–P63 (adolescence) & P56–P91 (adulthood)	• Start behavioral tests: P64 (adolescent group) P92 (adult group) • Body weight: every week during SIS • Food intake: daily during SIS • Brain collection: P64 (adolescent group) P92 (adult group)	• Fear-potentiated startle: (adolescence) SIS < CON(adulthood) SIS > CON	• Body weight gain: (adolescence/adulthood) SIS < CON • Food intake: (adolescence/adulthood) SIS < CON • Neuron morphology: (dendritic field BLA neurons): (adolescence) SIS < CON; (adulthood) SIS > CON (spine density BLA neurons): (adolescence) SIS < CON; (adulthood) SIS > CON • Neuron plasticity: (amygdalar FL-TrkB): (adolescence) SIS < CON; (adulthood) SIS > CON (amygdalar T-TrkB): (adolescence) SIS = CON; (adulthood) SIS > CON (amygdalar SNAP-25): (adolescence) SIS < CON; (adulthood) SIS = CON • Hippocampal BDNF: (adolescence) SIS < CON; (adulthood) SIS > CON

**McCormick et al. (2013a)**	• Long-Evans rats ♂ (Charles-River) • N = 80 (48 ♂: 24 SIS; 24 CON, 32 ♀ for sexual behavior observation)	• SIS^16D^: Pair (2/cage) changed daily for 16 days after 1 h isolation • P30–P45 (adolescence)	• Start behavioral tests: P86–P103 • Blood sample: P83 and P103 • Brain collection: P103	• Sexual behavior: (latency to ejaculate) SIS > CON (ejaculations) SIS < CON (sex score) SIS < CON	• Plasma testosterone: SIS < CON

**McCormick et al. (2013b)**	• Long-Evans rats ♀ (Charles-River) • N = 114 (57 SIS; 57 CON)	• SIS^16D^: Pair (2/cage) changed daily for 16 days after 1 h isolation • P30–P45 (adolescence) & P70–P85 (adulthood)	• Start behavioral tests: P46–P52 & P77–P83 (adolescent group) P86-92 & P117-123 (adult group)	• Fear conditioning: (freezing training) (acute/delayed, adolescence/adulthood) SIS = CON (freezing context test) (acute, adolescence) SIS < CON; (delayed, adolescence) SIS = CON; (acute/delayed, adulthood) SIS = CON (freezing cue test) (acute/delayed, adolescence/adulthood) SIS = CON (freezing extinction sessions) (acute/delayed, adolescence) SIS > CON; (acute/delayed, adulthood) SIS = CON • Sensitivity to foot shock: (delayed, adolescence) SIS = CON	

**Green et al. (2013)**	• Long-Evans rats ♂ (Charles-River) • N = 64 (32 SIS; 32 CON)	• SIS^16D^: Pair (2/cage) changed daily for 16 days after 1 h isolation • P30–P45 (adolescence)	• Start behavioral tests P70–P76	• OFT (latency to enter center): SIS < CON • Object recognition test: (latency to approach object) SIS < CON (interaction object) SIS < CON • Social interaction (time): SIS < CON	

**Green and McCormick et al. (2013)**	• Long-Evans rats ♂ (Charles-River) • N = 64 (17 SIS; 17 CON, 32 other)	• SIS^16D^: Pair (2/cage) changed daily for 16 days after 1 h isolation • P30–P45 (adolescence)	• Start behavioral tests: P85	• MWM: (distance travelled to platform) SIS = CON (improvement) SIS < CON • Spatial object location: (direct preference novel object) SIS = CON delayed preference novel object) SIS < CON	

**McCormick et al. (2012)**	• Long-Evans rats ♂ (Charles-River) • N = 116 (58 SIS; 58 CON)	• SIS^16D^: Pair (2/cage) changed daily for 16 days after 1 h isolation • P30–P45 (adolescence)	• Start behavioral tests: P46 & P70 • Brain collection: P46 & P74/P75	• Spatial location test: (investigation object novel location) (acute, delayed) SIS = CON (investigating object novel vs familiar location) (acute) SIS = CON (delayed) SIS < CON • Object recognition test (investigating objects): (acute, delayed) SIS = CON	• Hippocampal neurogenesis: (Ki67-ir): (acute, delayed) SIS = CON (DCX-ir): (acute, delayed) SIS > CON • Hippocampal plasticity: (Synaptophysin): (delayed) SIS = CON (CamKII, dorsal hippocampus): (delayed) SIS > CON (T286): (delayed) SIS < CON

**Morrissey et al. (2011)**	• Long-Evans rats ♂ (Charles-River) • N = 94 (47 SIS; 47 CON)	• SIS^16D^: Pair (2/cage) changed daily for 16 days after 1 h isolation • P30–P45 (adolescence)& P70–P85 (adulthood)	• Start behavioral tests: P46–P52 & P70–P76 (adolescent group) P86–P92 &P110–P116 (adult group)	• Fear conditioning: (freezing context test): (acute/delayed, adolescence) SIS < CON; (acute/delayed, adulthood) SIS = CON (freezing cue test): (acute/delayed, adolescence) SIS < CON; (acute/delayed, adulthood) SIS = CON	

**McCormick et al. (2010)**	• Long-Evans rats ♀ (Charles-River) • N = 52 (26 SIS; 26 CON)	• SIS^16D^: Pair (2/cage) changed daily for 16 days after 1 h isolation • P30–P45 (adolescence)	• Start behavioral tests: P46–P48 & P70–P72 • BrdU injections (10 SIS; 10 CON): P43–P45	• Spatial location test (investigation novel vs familiar object): (acute) SIS = CON; (delayed) SIS < CON	• Hippocampal cell proliferation (BrdU-ir DG): SIS < CON

**McCormick et al. (2008)**	• Long-Evans rats ♂ & ♀ (Charles-River) • N = 200 (100 ♂: 40 SIS; 40 CON, 20 other 100 ♀: 40 SIS; 40 CON, 20 other	• SIS^16D^: Pair (2/cage) changed daily for 16 days after 1 h isolation • P30–P45 (adolescence)	• Start behavioral tests: P45 & P70 • Blood sample: P45 & P70	• EPM: (open arms): (adolescence) SIS ♂ = CON ♂; SIS ♀ > CON ♀ (adulthood) SIS ♂ = CON ♂; SIS ♀ > CON ♀ (locomotor activity): (adolescence) SIS ♂ = CON ♂; SIS ♀ = CON ♀ (adulthood) SIS ♂ > CON ♂; SIS ♀ > CON ♀ (time center): (adolescence) SIS ♂ = CON ♂; SIS ♀ = CON ♀ (adulthood) SIS ♂ = CON ♂; SIS ♀ < CON ♀	• Plasma CORT: (adolescence) SIS ♂ < CON ♂; SIS ♀ = CON ♀ (adulthood) SIS ♂ = CON ♂; SIS ♀ = CON ♀

**Maslova et al. (2010)**	• Wistar rats ♂ (unknown) • N = 84 (30 SIS; 28 CON; 26 other)	• Other: Group (10/cage) composition changed daily for 42 days • P21–P63 (adolescence)	• Start behavioral tests: P64 & P134 • Brain/organ collection: P64 & P134 • Blood sample: P64 and P134	• Acoustic startle reaction: (acute) SIS = CON; (delayed) SIS < CON • EPM (open arms): (acute, delayed) SIS = CON • FST (passive drifting): (acute, delayed) SIS = CON	• Body weight: (acute, delayed) SIS = CON • Adrenal glands: (acute, delayed) SIS = CON • Systolic arterial blood pressure: (acute) SIS = CON; (delayed) SIS > CON • Plasma CORT: (acute, delayed) SIS = CON

**McCormick et al. (2007)**	• Long-Evans rats ♂ & ♀ (Charles-River) • N = 128 (44 ♂: 24 SIS; 20 CON, 44 ♀: 24 SIS; 20 CON, 40 other)	• SIS^16D^: Pair (2/cage) changed daily for 16 days after 1 h isolation • P30–P45 (adolescence)	• Body weight: P30 & P45 • Brain collection: P45 • Blood sample: P45		• Body weight: SIS ♀ = CON ♀; SIS ♂ < CON ♂ • Body weight gain: SIS ♀ = CON ♀; SIS ♂ < CON ♂ • Plasma CORT: SIS ♀ = CON ♀; SIS ♂ < CON ♂ • CRH mRNA in PVN: SIS ♀ > CON ♀; SIS ♂ > CON ♂

**Abbreviations**: ACTH: adrenocorticotropic hormone; BLA: basolateral amygdala; C1: cohort 1; C2: cohort 2; CFC: contextual fear conditioning; CON: Control; CORT: corticosterone; CPP: conditioned place preference; CRH: corticotrophin releasing hormone; EPM: elevated plus maze; MWM: Morris water maze; ns: not specified; NSF: novelty suppressed feeding; OD: optical density; OFT: open field test; PPI: pre-pulse inhibition task; SIS: Social Instability Stressed; SPT: sucrose preference test; TST: tail suspension test.

**Table:** 

Article	Sample	SIS protocol	Acute/Delayed	Behavioral results	Physiological results
**Wang et al. (2019)**	• C57BL/6 mice ♂ (Vital River) • N = 64 (32 SIS; 32 CON)	• SIS^49D^: Group (4/cage) composition changed twice weekly for 49 days • P29-77 (adolescence)	• Start behavioral tests: ± P456	• SPT (sucrose preference): SIS < CON • TST (latency to immobility): SIS < CON • OFT (total distance travelled): SIS = CON • PPI: SIS = CON	

**Yohn et al. (2019)**	• C57BL/6J mice ♂ & ♀ (unknown) • Total sample size: 181 (45 ♂: 25 SIS; 20 CON 48 ♀: 28 SIS; 20 CON, 88 other)	• SIS^49D^: Group (3–5/cage) composition changed every 3 days for 49 days • P56-105 (adulthood)	• Start behavioral tests: P105–P112 • Brain collection: P119 • Blood sample: P98	• OFT (center): SIS ♂ < CON ♂; SIS ♀ < CON ♀ • Light-Dark Box (light): SIS ♂ < CON ♂; SIS ♀ < CON ♀ • EPM (open arms): SIS ♂ = CON ♂; SIS ♀ = CON ♀ • FST (immobility time): SIS ♂ = CON ♂; SIS ♀ = CON ♀ • NSF (latency to eat): SIS M > CON M; SIS ♀ > CON ♀	• Adult hippocampal neurogenesis: (Ki67+ cells): SIS ♂ = CON ♂; SIS ♀ = CON ♀ (DCX + cells): SIS ♂ = CON ♂; SIS ♀ < CON ♀ • Plasma CORT: SIS ♂ > CON ♂; SIS ♀ > CON ♀

**Dadomo et al. (2018)**	• CD1 mice ♀ (Charles River) • N = 44 (11 SIS; 11 CON, 22 other)	• Other: Group (3/cage) composition changed daily for 28 days • P70–P98 (adulthood)	• Start behavioral tests: during SIS • Blood sample: P99 & P100 • Food intake: every two weeks • Organ collection: P100	• OFT (ns): SIS = CON • EPM (ns): SIS = CON • SPT (sucrose consumption): SIS < CON	• Body weight gain: SIS < CON • Food intake: SIS < CON • Adrenal glands weight: SIS = CON • Plasma CORT: SIS = CON • Plasma ACTH: SIS = CON

**de Lima and Massoco (2017)**	• Balb/c mice ♂ (unknown) • N = 16 (8 SIS; 8 CON)	• Other: Pair (2/cage) changed daily for 11 days after 1 h isolation • P30–P40 (adolescence)	• Start behavioral tests: P60–P62 • Body weight: not specified	• OFT: (total distance) SIS = CON (center) SIS = CON • Light-Dark Box: (risk assessment) SIS = CON (light chamber) SIS = CON • TST: (latency to immobility) SIS < CON (frequency of immobility) SIS > CON (immobility time) SIS > CON	• Body weight: SIS = CON

**Boleij et al. (2014)**	• CD1 mice ♂ (Charles River) • N = 48 (32 SIS; 16 CON)	• SIS^49D^: Group (4/cage) composition changed twice weekly for 49 days • P35–P84 (adolescence)	• Start behavioral tests: P91 • Blood sample: P35 & P84 & P91 • Brain/organ collection: P84 & P91 • Fur state scored during SIS	• Modified hole board (exploration): SIS = CON • Locomotor activity (immobility time) SIS = CON	• Body weight: SIS < CON • Body weight gain: SIS < CON • Fur state: SIS < CON • Adrenal glands: SIS = CON • *Nr3c1* expression: (CA1): SIS < CON (DG): SIS < CON • *Nr3c2* expression: (CA2) SIS > CON (DG) SIS = CON

**Saavedra-Rodríguez and Feig (2013)**	• CD1 mice (Charles-River) ♂ & ♀ • N = not specified (>22)	• SIS^49D^: Group (4/cage) composition changed twice weekly for 49 days • P27-76 (adolescence)	• Start behavioral tests: ±P136	• EPM (open arms): SIS ♂ = CON ♂; SIS ♀ < CON ♀ • OFT (locomotor adaptation): SIS ♂ = CON ♂; SIS ♀ < CON ♀ • Social interaction with juvenile (time): SIS ♂ < CON ♂; SIS ♀ < CON ♀ • Sociability (time social chamber): SIS ♂ = CON ♂; SIS ♀ = CON ♀ • Social novelty test (time novel social chamber): SIS ♂ < CON ♂; SIS ♀ < CON ♀	

**Scharf et al. (2013)**	• CD1 mice ♂ (Charles-River) • N = 164 [46 SIS; 50 CON, vehicle (VEH); 34 SIS; 34 CON, paroxetine (PAR)]	• SIS^49D^: Group (4/cage) composition changed twice weekly for 49 days • P28–P77 (adolescence)	• Start behavioral experiments: P112 & P445; 15moa (aged) • Body weight: P28, P77, P112 & P445; 15 moa (aged) • Blood sample: P77, P112 & P445; 15 moa (aged) • Brain/organ collection: P112 & P445; 15 moa (aged)	• EPM (open arms): (adult) SIS = CON • OFT (distance travelled first 30 s): (adult) SIS < CON • OFT (distance travelled last 30 s): (adult) SIS^VEH^ > CON^VEH^ (adult) SIS^PAR^ > CON^VEH^ • Social interaction with male DBA mouse (time): (adult) SIS = CON • TST (immobility time): (adult) SIS = CON • EPM (open arms): (aged) SIS = CON	• Body weight: (adult) SIS = CON (aged) SIS = CON • Plasma CORT: (adolescence) SIS > CON (adult) SIS > CON (aged) SIS = CON • Thymus: (adult) SIS < CON (aged) ns • Adrenal: (adult) SIS = CON (aged) SIS < CON • Gene expression PVN (AVP mRNA): (adult) SIS = CON (aged) SIS = CON • CRH mRNA in PVN (adult) SIS = CON (aged) SIS < CON • *Nr3c1* expression (CA1/PVN): (adult) SIS = CON (aged) SIS = CON • *Nr3c2* expression (PVN/CA1): (adult) SIS = CON (aged) SIS = CON

**Schmidt et al. (2010a)**	• CD1 mice ♀ (Charles-River) • N = 64 (32 SIS; 32 CON)	• SIS^49D^: Group (4/cage) composition changed twice weekly for 49 days • P31–P80 (adolescence)	• Start behavioral tests: P81–P83 • Blood sample: P80 • Brain/organ collection: P80	• OFT (distance travelled): SIS = CON • NSF (time till consumption): SIS > CON • EPM: (time open arms) SIS = CON (entries open arms) SIS = CON	• Body weight: SIS = CON • Adrenal glands: SIS > CON • Thymus: SIS < CON • Plasma CORT morning: SIS > CON • Plasma ACTH: SIS = CON • CORT/ACTH ratio: SIS > CON • *Nr3c2* expression: (CA2): SIS < CON (DG OD): SIS < CON (CA2/CA3 OD): SIS = CON • *Nr3c1* expression: (CA1): SIS = CON (DG): SIS = CON • Gene expression in PVN: (CRH mRNA): SIS < CON (AVP mRNA): SIS = CON (*Nr3c1*): SIS < CON

**Schmidt et al. (2010b)**	• CD1 mice ♂ (Charles-River) • N = 200 (160 SIS; 40 CON)	• SIS^49D^: Group (4/cage) composition changed twice weekly for 49 days • P29/31-P78/80 (adolescence)	• Start behavioral tests: P114/P116 – P119/P121 • Brain/organ collection: P114/P116 • Blood sample: P28/30 & P79/81 & P114/P116	• OFT: (locomotor activity) SIS = CON (distance first 3 min) SIS > CON • EPM: (open arms) SIS = CON (entries open arms) SIS = CON • TST: (immobility time) SIS = CON (immobile episodes) SIS = CON	• Body weight: (acute, delayed) SIS = CON • Body weight gain: (acute, delayed) SIS = CON • Adrenal glands: (acute, delayed) SIS > CON • Plasma CORT: (acute, delayed) SIS > CON • CRH mRNA in PVN: SIS = CON • *Nr3c2* expression (CA1/CA2/CA3/DG):SIS = CON • *Nr3c1* expression (CA1/DG/PVN):SIS = CON

**Sterlemann et al. (2010)**	• CD1 mice ♂ (Charles-River) • N = 40 (20 SIS; 22 CON)	• SIS^49D^: Group (4/cage) composition changed twice weekly for 49 days • P31/33-P80/P82 (adolescence)	• Start behavioral tests: P445/447; 15 moa • Brain/organ collection: P459/P461	• MWM: (time first two days):SIS = CON (time third day): SIS > CON • Y-Maze (novel arm exploration): SIS < CON • Social discrimination (exploration): SIS = CON • Object recognition test (exploration): SIS = CON	• Synaptic plasticity (LTP CA1): SIS < CON (Synaptophysin DG): SIS < CON; (Synaptophysin CA1/CA3): SIS = CON • Hippocampal BDNF: SIS < CON

**Chatterjee et al. (2009)**	• SJL mice ♂ (Taconic Farms) • N = 36 (24 SIS; 15 CON)	• Other: Pair (2/cage) changed daily for 19 days • P42–P61 (adulthood)	• Body weight: every 6 days • Brain/organ collection: P62 • Blood sample: P62		• Body weight gain: SIS < CON • Mean weight: SIS < CON • Serum CORT: SIS > CON • Adrenal BK channel mRNA: (Slo-α): SIS > CON (STREX): SIS > CON (β2): SIS = CON (β4): SIS = CON • Pituitary BK channel mRNA: (Slo-α): SIS > CON (STREX): SIS = CON (β2): SIS > CON (β4): SIS > CON

**Schmidt et al. (2009)**	• CD1 mice ♂ (Charles-River) • N = 42 (20 SIS; 22 CON)	• SIS^49D^: Group (4/cage) composition changed twice weekly for 49 days • P31/33-P80/P82 (adolescence)	• Body weight: every two weeks • Start behavioral tests: P445/447; 15 moa	• OFT (locomotor activity): SIS = CON	• Body weight: SIS = CON • Food intake: SIS = CON • Plasma CORT (morning): SIS = CON • Fasting glucose levels: SIS = CON • MR imaging (intra-abdominal fat distribution): (subcutaneous fat): SIS < CON (visceral fat): SIS = CON (visceral-to-subcutaneous fat ratio): SIS > CON

**Sterlemann et al. (2008)**	• CD1 mice ♂ (Charles-River) • N = 108 (64 SIS; 64 CON)	• SIS^49D^: Group (4/cage) composition changed twice weekly for 49 days • P31/33-P80/82 (adolescence)	• Start behavioral tests: P87/89-P91/P93 & P445; 15 moa • Blood was collected from tail at P81/83 and ±P420 • Brain/organs collection: P91/P93 & ±P420	• OFT (total distance): (acute, delayed) SIS = CON • NSF (time till consumption): (acute) SIS > CON; (delayed) SIS = CON • EPM: (time open arms): (acute, delayed) SIS = CON (entries open arms): (acute) SIS < CON; (delayed) SIS = CON (nr of head dips): (acute) SIS = CON; (delayed) SIS < CON	• Body weight: (acute, delayed) SIS = CON • Adrenal glands: (acute) SIS > CON; (delayed) SIS = CON • Thymus: (acute) SIS < CON • Plasma CORT: (acute) SIS > CON; (acute, evening): SIS < CON; (delayed) SIS = CON • Plasma ACTH: (acute, morning) SIS = CON • CORT/ACTH ratio: (acute) SIS > CON • *Nr3c2* expression: (CA1/CA2/DG): (acute) SIS < CON; (delayed) SIS = CON (CA3): (acute) SIS < CON; (delayed) SIS < CON • *Nr3c1* expression (CA1): (acute) SIS < CON; (delayed) SIS = CON • Gene expression PVN (AVP mRNA): (acute) SIS > CON; (delayed) SIS = CON

**Schmidt et al. (2007)**	• CD1 mice ♂ (Charles-River) • N = 96 (48 SIS; 48 CON)	• SIS^49D^: Group (4/cage) composition changed twice weekly for 49 days • P31/33-P80/82 (adolescence)	• Start behavioral tests: P87/89-P91/P93 • Blood sample: P81/83 & P91/P93 • Brain/organ collection: P91/P93	• OFT (locomotor adaptation): SIS < CON • NSF (time till consumption): SIS > CON • EPM: (time open arms) SIS = CON (entries open arms) SIS < CON • Social interactions: (aggressive attacks) SIS > CON (latency aggressive attack) SIS < CON	• Body weight: SIS = CON • Body weight gain: SIS = CON • Plasma CORT: (acute) SIS > CON; (delayed) SIS = CON • Plasma ACTH: (delayed, morning) SIS < CON (delayed, evening): SIS = CON • CORT/ACTH ratio: SIS > CON • Body weight: SIS = CON • Adrenal glands: SIS > CON • Thymus: SIS < CON • Fur state: SIS < CON • *Nr3c2* expression (CA2): SIS < CON • *Nr3c1* expression (CA1): SIS < CON

**Abbreviations**: ACTH: adrenocorticotropic hormone; BLA: basolateral amygdala; C1: cohort 1; C2: cohort 2; CFC: contextual fear conditioning; CON: Control; CORT: corticosterone; CPP: conditioned place preference; CRH: corticotrophin releasing hormone; EPM: elevated plus maze; moa: months of age; MWM: Morris water maze; ns: not specified; NSF: novelty suppressed feeding; OD: optical density; OFT: open field test; PPI: pre-pulse inhibition task; SIS: Social Instability Stress; SPT: sucrose preference test; TST: tail suspension test.
