# Supplementary material for: The social instability stress paradigm in rat and mouse: A systematic review of protocols, limitations, and recommendations
Source: Neurobiol Stress. 2021 Oct 16;15:100410. doi: 10.1016/j.ynstr.2021.100410 (PMC8648958; doi:10.1016/j.ynstr.2021.100410)
Supplement: Multimedia component 1 [file mmc1.docx]

**Full-text articles excluded (n=45)**

Combined with other stressor (n=18)

1. Baranyi, J., Bakos, N., & Haller, J. (2005). Social instability in female rats: the relationship between stress-related and anxiety-like consequences. *Physiology & behavior*, *84*(4), 511-518.
2. Labaka, A., Gómez-Lázaro, E., Vegas, O., Pérez-Tejada, J., Arregi, A., & Garmendia, L. (2017). Reduced hippocampal IL-10 expression, altered monoaminergic activity and anxiety and depressive-like behavior in female mice subjected to chronic social instability stress. *Behavioural brain research*, *335*, 8-18.
3. Herzog, C. J., Czéh, B., Corbach, S., Wuttke, W., Schulte-Herbrüggen, O., Hellweg, R., ... & Fuchs, E. (2009). Chronic social instability stress in female rats: a potential animal model for female depression. *Neuroscience*, *159*(3), 982-992.
4. Jarcho, M. R., Massner, K. J., Eggert, A. R., & Wichelt, E. L. (2016). Behavioral and physiological response to onset and termination of social instability in female mice. *Hormones and behavior*, *78*, 135-140.
5. Dickson, D. A., Paulus, J. K., Mensah, V., Lem, J., Saavedra-Rodriguez, L., Gentry, A., ... & Feig, L. A. (2018). Reduced levels of miRNAs 449 and 34 in sperm of mice and men exposed to early life stress. *Translational Psychiatry*, *8*(1), 1-10.
6. Øines, E., Murison, R., Mrdalj, J., Grønli, J., & Milde, A. M. (2012). Neonatal maternal separation in male rats increases intestinal permeability and affects behavior after chronic social stress. *Physiology & behavior*, *105*(4), 1058-1066.
7. Zoladz, P. R., D'Alessio, P. A., Seeley, S. L., Kasler, C. D., Goodman, C. S., Mucher, K. E., ... & Rorabaugh, B. R. (2019). A predator-based psychosocial stress animal model of PTSD in females: Influence of estrous phase and ovarian hormones. *Hormones and behavior*, *115*, 104564.
8. Zoladz, P. R., Conrad, C. D., Fleshner, M., & Diamond, D. M. (2008). Acute episodes of predator exposure in conjunction with chronic social instability as an animal model of post-traumatic stress disorder. *Stress*, *11*(4), 259-281.
9. Zoladz, P. R., Fleshner, M., & Diamond, D. M. (2013). Differential effectiveness of tianeptine, clonidine and amitriptyline in blocking traumatic memory expression, anxiety and hypertension in an animal model of PTSD. *Progress in Neuro-Psychopharmacology and Biological Psychiatry*, *44*, 1-16.
10. Roth, T. L., Zoladz, P. R., Sweatt, J. D., & Diamond, D. M. (2011). Epigenetic modification of hippocampal Bdnf DNA in adult rats in an animal model of post-traumatic stress disorder. *Journal of psychiatric research*, *45*(7), 919-926.
11. Seetharaman, S., Fleshner, M., Park, C. R., & Diamond, D. M. (2016). Influence of daily social stimulation on behavioral and physiological outcomes in an animal model of PTSD. *Brain and behavior*, *6*(5), e00458.
12. Zoladz, P. R., & Diamond, D. M. (2016). Predator-based psychosocial stress animal model of PTSD: Preclinical assessment of traumatic stress at cognitive, hormonal, pharmacological, cardiovascular and epigenetic levels of analysis. *Experimental neurology*, *284*, 211-219.
13. Zoladz, P. R., Fleshner, M., & Diamond, D. M. (2012). Psychosocial animal model of PTSD produces a long-lasting traumatic memory, an increase in general anxiety and PTSD-like glucocorticoid abnormalities. *Psychoneuroendocrinology*, *37*(9), 1531-1545.
14. Zoladz, P. R., & Diamond, D. (2016). Psychosocial predator stress model of PTSD based on clinically relevant risk factors for trauma-induced psychopathology. *Posttraumatic Stress Disorder: From Neurobiology to Treatment*, *125*, 125-143.
15. Zoladz, P. R., Park, C. R., Fleshner, M., & Diamond, D. M. (2015). Psychosocial predator-based animal model of PTSD produces physiological and behavioral sequelae and a traumatic memory four months following stress onset. *Physiology & behavior*, *147*, 183-192.
16. Rorabaugh, B. R., Krivenko, A., Eisenmann, E. D., Bui, A. D., Seeley, S., Fry, M. E., ... & Zoladz, P. R. (2015). Sex-dependent effects of chronic psychosocial stress on myocardial sensitivity to ischemic injury. *Stress*, *18*(6), 645-653.
17. Dametto, M., Suchecki, D., Bueno, O. F., Moreira, K. M., Tufik, S., & Oliveira, M. G. M. (2002). Social stress does not interact with paradoxical sleep deprivation-induced memory impairment. *Behavioural brain research*, *129*(1-2), 171-178.
18. Suchecki, D., & Tufik, S. (2000). Social stability attenuates the stress in the modified multiple platform method for paradoxical sleep deprivation in the rat. *Physiology & behavior*, *68*(3), 309-316.

Pharmacological drug/substance supplementation (n=13)

1. Al-Rahbi, B., Zakaria, R., Othman, Z., Hassan, A., Ismail, Z. I. M., & Muthuraju, S. (2014). Tualang honey supplement improves memory performance and hippocampal morphology in stressed ovariectomized rats. *Acta Histochemica*, *116*(1), 79-88.
2. Nowacka-Chmielewska, M. M., Kasprowska, D., Paul-Samojedny, M., Bielecka-Wajdman, A. M., Barski, J. J., Małecki, A., & Obuchowicz, E. (2017). The effects of desipramine, fluoxetine, or tianeptine on changes in bulbar BDNF levels induced by chronic social instability stress and inflammation. *Pharmacological Reports*, *69*(3), 520-525.
3. Haller, J., Baranyi, J., Bakos, N., & Halász, J. (2004). Social instability in female rats: effects on anxiety and buspirone efficacy. *Psychopharmacology*, *174*(2), 197-202.
4. Lemaire, V., Le Moal, M. I. C. H. E. L., & Mormede, P. (1993). Regulation of catecholamine-synthesizing enzymes in adrenals of Wistar rats under chronic stress. *American Journal of Physiology-Regulatory, Integrative and Comparative Physiology*, *264*(5), R957-R962.
5. Provensi, G., Schmidt, S. D., Boehme, M., Bastiaanssen, T. F., Rani, B., Costa, A., ... & Passani, M. B. (2019). Preventing adolescent stress-induced cognitive and microbiome changes by diet. *Proceedings of the National Academy of Sciences*, *116*(19), 9644-9651.
6. Nowacka, M. M., Paul-Samojedny, M., Bielecka, A. M., Plewka, D., Czekaj, P., & Obuchowicz, E. (2015). LPS reduces BDNF and VEGF expression in the structures of the HPA axis of chronic social stressed female rats. *Neuropeptides*, *54*, 17-27.
7. McCormick, C. M., & Ibrahim, F. N. (2007). Locomotor activity to nicotine and Fos immunoreactivity in the paraventricular nucleus of the hypothalamus in adolescent socially-stressed rats. *Pharmacology Biochemistry and Behavior*, *86*(1), 92-102.
8. Cohn, D. W. H., Gabanyi, I., Kinoshita, D., & de Sá-Rocha, L. C. (2012). Lipopolysaccharide administration in the dominant mouse destabilizes social hierarchy. *Behavioural processes*, *91*(1), 54-60.
9. Syme, L. A., & Syme, G. J. (1974). Group instability and the social response to methamphetamine. *Pharmacology Biochemistry and Behavior*, *2*(6), 851-854.
10. Al-Rahbi, B., Zakaria, R., Othman, Z., Hassan, A., & Ahmad, A. H. (2014). Enhancement of BDNF concentration and restoration of the hypothalamic-pituitary-adrenal axis accompany reduced depressive-like behaviour in stressed ovariectomised rats treated with either Tualang honey or estrogen. *The Scientific World Journal*, *2014*.
11. Hodges, T. E., Eltahir, A. M., Patel, S., Bredewold, R., Veenema, A. H., & McCormick, C. M. (2019). Effects of oxytocin receptor antagonism on social function and corticosterone release after adolescent social instability in male rats. *Hormones and behavior*, *116*, 104579.
12. Nowacka, M. M., Paul-Samojedny, M., Bielecka, A. M., & Obuchowicz, E. (2014). Chronic social instability stress enhances vulnerability of BDNF response to LPS in the limbic structures of female rats: A protective role of antidepressants. *Neuroscience research*, *88*, 74-83.
13. Nowacka‑Chmielewska, M. M., Paul‑Samojedny, M., Bielecka‑Wajdman, A. M., Barski, J. J., & Obuchowicz, E. (2017). Alterations in VEGF expression induced by antidepressant drugs in female rats under chronic social stress. *Experimental and therapeutic medicine*, *13*(2), 723-730.

No stress parameter measured (n=4)

1. McCormick, C. M., Cameron, N. M., Thompson, M. A., Cumming, M. J., Hodges, T. E., & Langett, M. (2017). The sexual preference of female rats is influenced by males' adolescent social stress history and social status. *Hormones and behavior*, *89*, 30-37.
2. Hodges, T. E., Louth, E. L., Bailey, C. D., & McCormick, C. M. (2019). Adolescent social instability stress alters markers of synaptic plasticity and dendritic structure in the medial amygdala and lateral septum in male rats. *Brain Structure and Function*, *224*(2), 643-659.
3. Cumming, M. J., Thompson, M. A., & McCormick, C. M. (2014). Adolescent social instability stress increases aggression in a food competition task in adult male Long‐Evans rats. *Developmental Psychobiology*, *56*(7), 1575-1588.
4. Mugford, R. A. (1973). Intermale fighting affected by home-cage odors of male and female mice. *Journal of comparative and physiological psychology*, *84*(2), 289.

Surgical removal and/or implantation (n=7)

1. Lemaire, V., Taylor, G. T., & Mormède, P. (1997). Adrenal axis activation by chronic social stress fails to inhibit gonadal function in male rats. *Psychoneuroendocrinology*, *22*(8), 563-573.
2. Lemaire, V., Deminière, J. M., & Mormède, P. (1994). Chronic social stress conditions differentially modify vulnerability to amphetamine self-administration. *Brain research*, *649*(1-2), 348-352.
3. Haller, J., Fuchs, E., Halász, J., & Makara, G. B. (1999). Defeat is a major stressor in males while social instability is stressful mainly in females: towards the development of a social stress model in female rats. *Brain research bulletin*, *50*(1), 33-39.
4. Leedy, G. M., Barrows, L. F., & Clark, S. (2013). Effects of social housing on hippocampal dendrites and behavior in ovariectomized rats. *Brain research bulletin*, *92*, 69-75.
5. Al-Rahbi, B., Zakaria, R., Othman, Z., Hassan, A., Muthuraju, S., & Wan Mohammad, W. M. Z. (2013). Mood and memory function in ovariectomised rats exposed to social instability stress. *BioMed Research International*, *2013*.
6. Al-Rahbi, B., Zakaria, R., Muthuraju, S., Othman, Z., & Hassan, A. (2013). Preliminary study: effects of social instability stress on depressive behaviours in ovariectomised rats. *The Malaysian journal of medical sciences: MJMS*, *20*(2), 35.
7. Lemaire, V., & Mormède, P. (1995). Telemetered recording of blood pressure and heart rate in different strains of rats during chronic social stress. *Physiology & behavior*, *58*(6), 1181-1188.

Transgenic line (n=1)

1. Opendak, M., Offit, L., Monari, P., Schoenfeld, T. J., Sonti, A. N., Cameron, H. A., & Gould, E. (2016). Lasting adaptations in social behavior produced by social disruption and inhibition of adult neurogenesis. *Journal of Neuroscience*, *36*(26), 7027-7038.

No control group (n=2)

1. Avgustinovich, D. F., & Kovalenko, I. L. (2004). Long-term isolated housing causes anxiety in C57BL/6J female mice. *Rossiiskii fiziologicheskii zhurnal imeni IM Sechenova*, *90*(3), 351-360.
2. Branchi, I., Santarelli, S., D'Andrea, I., & Alleva, E. (2013). Not all stressors are equal: early social enrichment favors resilience to social but not physical stress in male mice. *Hormones and behavior*, *63*(3), 503-509.
